# Advanced patient-specific microglia cell models for pre-clinical studies in Alzheimer’s disease

**DOI:** 10.1186/s12974-024-03037-3

**Published:** 2024-02-15

**Authors:** Carla Cuní-López, Romal Stewart, Lotta E. Oikari, Tam Hong Nguyen, Tara L. Roberts, Yifan Sun, Christine C. Guo, Michelle K. Lupton, Anthony R. White, Hazel Quek

**Affiliations:** 1 Mental Health and Neuroscience Department, QIMR Berghofer Medical Research Institute, Herston, QLD 4006 Australia; 2 Faculty of Medicine, The University of Queensland, Herston, QLD 4006 Australia; 3 School of Biomedical Sciences, The University of Queensland, Lucia, QLD 4072 Australia; 4 School of Biomedical Sciences, Queensland University of Technology, Brisbane City, QLD 4000 Australia; 5 UQ Centre for Clinical Research, The University of Queensland, Brisbane City, QLD 4029 Australia; 6 Scientific Services, QIMR Berghofer Medical Research Institute, Herston, QLD 4006 Australia; 7 Ingham Institute for Applied Medical Research and School of Medicine, Western Sydney University, Liverpool, NSW 2170 Australia; 8 Present Address: ActiGraph LLC, Pensacola, FL 32502 USA

**Keywords:** Microglia, Monocytes, Patient, Alzheimer’s disease, 3D cell modeling, Drugs

## Abstract

**Background:**

Alzheimer’s disease (AD) is an incurable neurodegenerative disorder with a rapidly increasing prevalence worldwide. Current approaches targeting hallmark pathological features of AD have had no consistent clinical benefit. Neuroinflammation is a major contributor to neurodegeneration and hence, microglia, the brain’s resident immune cells, are an attractive target for potentially more effective therapeutic strategies. However, there is no current in vitro model system that captures AD patient-specific microglial characteristics using physiologically relevant and experimentally flexible culture conditions.

**Methods:**

To address this shortcoming, we developed novel 3D Matrigel-based monocyte-derived microglia-like cell (MDMi) mono-cultures and co-cultures with neuro-glial cells (ReNcell VM). We used single-cell RNA sequencing (scRNAseq) analysis to compare the transcriptomic signatures of MDMi between model systems (2D, 3D and 3D co-culture) and against published human microglia datasets. To demonstrate the potential of MDMi for use in personalized pre-clinical strategies, we generated and characterized MDMi models from sixteen AD patients and matched healthy controls, and profiled cytokine responses upon treatment with anti-inflammatory drugs (dasatinib and spiperone).

**Results:**

MDMi in 3D exhibited a more branched morphology and longer survival in culture compared to 2D. scRNAseq uncovered distinct MDMi subpopulations that exhibit higher functional heterogeneity and best resemble human microglia in 3D co-culture. AD MDMi in 3D co-culture showed altered cell-to-cell interactions, growth factor and cytokine secretion profiles and responses to amyloid-β. Drug testing assays revealed patient- and model-specific cytokine responses.

**Conclusion:**

Our study presents a novel, physiologically relevant and AD patient-specific 3D microglia cell model that opens avenues towards improving personalized drug development strategies in AD.

**Supplementary Information:**

The online version contains supplementary material available at 10.1186/s12974-024-03037-3.

## Background

Alzheimer’s disease (AD) is an age-related neurodegenerative disorder involving progressive impairment of cognitive functions. Although AD—together with other neurodegenerative diseases—is predicted to become the second leading cause of death in the next 20 years, no prevention strategies or cure exist [1]. The most characteristic neuropathological hallmarks of AD brains include extracellular deposits of misfolded amyloid-β (Aβ) protein and intracellular neurofibrillary tangles of hyperphosphorylated tau protein. For decades, reducing protein aggregation has been the main therapeutic goal in AD, but this strategy has yielded poor clinical outcomes with questioned efficacy. For example, aducanumab and lecanemab have been the first approved amyloid-targeting treatments in nearly 20 years, but their safety and cognitive benefits are debated [2, 3].

Chronic neuroinflammation is a critical component in the progression of many neurodegenerative diseases, including AD [4]. Key neuroinflammatory effectors are microglia, the resident immune modulators of the brain. Microglial function is controlled by variants in AD risk genes (*e.g.*, *TREM2*, *APOE*, *CLU, CD33*, *PILRB* and *PLCG2*) [5]. Such variants modulate microglial responses to AD pathological events [6–8], thus underlying the diversity of clinical phenotypes observed among patients. Hence, microglia represent a promising candidate for personalized, targeted therapeutics for AD.

Current AD microglia in vitro model systems (reviewed in [9, 10]) lack either clinical relevance or physiological complexity, thereby affecting translatability of drug outcomes into the clinic. For example, murine microglia lack the ability to fully recapitulate disease phenotype due to limited resemblance of immune functions and aging processes between mice and humans [11–14]. Human immortalized microglia cell lines are genetically and functionally very different from in vivo microglia [15–17]. Post-mortem primary microglia isolated from AD patients rapidly lose microglial signatures upon removal from the brain environment [15]. Conversely, human induced pluripotent stem cell (hiPSC)-derived microglia allow for the generation of a clinically relevant, patient-specific microglia platform. However, establishing hiPSC-derived microglia requires costly, long and technically challenging protocols that result in variable differentiation efficiencies [18] and loss of patient-specific traits upon reprogramming [19].

The monocyte-derived microglia-like cell (MDMi) model system addresses the shortcomings of the above models and provides a cost-effective approach for the rapid generation of personalized microglia-like cell cultures from living patients. This method has been previously applied by us and others using ex vivo blood-derived monocytes from schizophrenia [20, 21], Nasu–Hakola disease [22] and amyotrophic lateral sclerosis (ALS) [23] patients, demonstrating disease-associated states in patient-derived MDMi. In addition to their controlled genetic background, MDMi are readily available and yield mature microglia-like cells in a short time frame, thus allowing for the study of microglia from large patient cohorts [24, 25].

Microglial identity is driven by the multicellular milieu and three-dimensional (3D) network of macromolecules present in the brain. The lack of such structures in traditional two dimensional (2D) culture conditions greatly abrogate the ability of 2D in vitro models to replicate mature microglial characteristics, which include a ramified morphology and the ability to efficiently survey the brain parenchyma through the upregulation of key markers (*e.g*., TMEM119, P2RY12 and TREM2) [26, 27]. The use of 3D in vitro culture techniques and co-cultures with neuro-glial cells, which mimic the cues supporting microglial development in vivo, can increase the physiological relevance of the MDMi model. Indeed, an improved in vitro disease modeling capacity of 3D culture systems was demonstrated by the development of a complete AD pathological cascade in 3D but not in 2D [28, 29]. However, no 3D in vitro model of AD has yet incorporated patient-derived microglial cells in a highly reproducible and experimentally flexible 3D cell culture system [30].

In this study, we generated for the first time 3D patient-specific MDMi models from living AD patients. These 3D hydrogel-based MDMi models are consistent and easy to generate and allow for the establishment of 3D MDMi co-cultures with human neuro-glial cells. We identified that 3D MDMi exhibit enhanced microglia-like features and AD-specific changes in the patient-derived models. To test the potential applicability of 3D MDMi platforms in a drug screening setting, we compared drug responses in MDMi between the 2D, 3D and 3D co-culture models. Together, the utility of the patient 3D MDMi models presented here opens new avenues for more predictable and personalized patient in vitro microglia-like cell models to test candidate therapeutics.

## Materials and methods

### Study cohorts

**Fig. 1:**
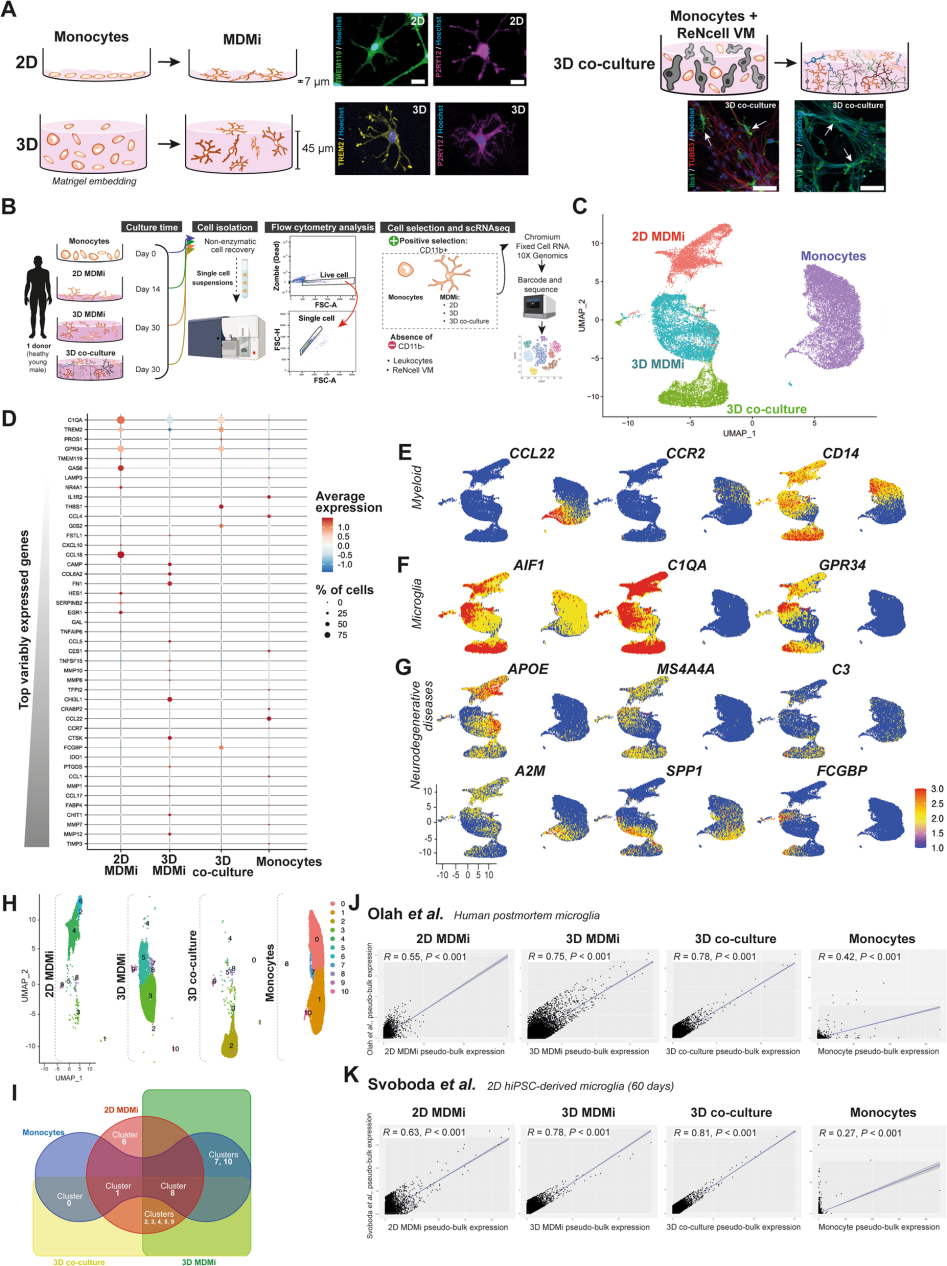
Single cell RNAseq reveals model-specific transcriptomic signatures in MDMi and resemblance to post-mortem and hiPSC-derived microglia. **A** Schematic illustrations of the differentiation of monocytes into MDMi as mono-cultures in 2D and 3D, and as 3D co-cultures with ReNcell VM. Immunofluorescence of MDMi in the different culture models with immunostaining against Tmem119 and P2ry12 in 2D; Trem2 and P2ry12 in 3D; and Iba1 (arrows) in 3D co-culture. ReNcell VM were stained for the neuron marker β3-tubulin (Tubb3) and the astrocyte marker Gfap. Scale bars, 100 μm. **B** Schematic of the methodology employed for single cells transcriptome analysis in monocytes and MDMi (2D, 3D, and 3D co-culture). Monocytes and MDMi from a single healthy donor were cultured, FACs-sorted for CD11b, fixed according to Chromium fixed RNA kit (10X Genomics), sequenced using Illumina NextSeq-2000 and analyzed (detailed protocol can be found in Methods). **C** UMAP plot showing the clustering of monocyte and MDMi (2D, 3D and 3D co-culture) single cell transcriptomic signatures. The total number of cells analyzed was: 9521 monocytes; 5608 MDMi in 2D; 5667 MDMi in 3D and 4543 MDMi in 3D co-culture. **D** Top 45 variable genes, with the most variable genes listed at the bottom and the most constant genes at the top of the list. Each row represents the level of expression of a selected key gene. The color of the dot represents the average expression z-score of the cells within the given cluster, while the size of the dot represents the % of cells within that cluster. **E–G** Combined UMAP plots of monocytes and MDMi (2D, 3D and 3D co-culture) show the expression of selected key myeloid, microglia and neurodegenerative disease-related genes. Each dot represents a cell and the normalized gene expression levels of the selected genes for each cell. The color gradient bar represents log-transformed expression values, with red and blue indicating maximum and minimum expression, respectively. **H** Distribution of clusters in monocytes and MDMi (2D, 3D and 3D co-culture) to find shared or unique pathways across the models. Pathways of these 10 clusters and can be found in Additional file 1: Fig. S7. **I** Venn diagram illustrating shared and/or unique clusters among all model systems. **J, K** Pseudo-bulk gene expression in monocytes and MDMi (2D, 3D, 3D co-culture) compared to human microglia. Human microglial genes were selected from post-mortem brain datasets (Olah et al.: Clusters 1–9) and Day 60 hiPSC-derived microglia (Svoboda et al.). Correlation tests were based on the following number of genes 13,798 (monocytes); 14,480 (2D); 14,220 (3D); 14,216 (3D co-culture). Spearman correlation coefficient (R) was used to examine the degree of correlation

**Fig. 2:**
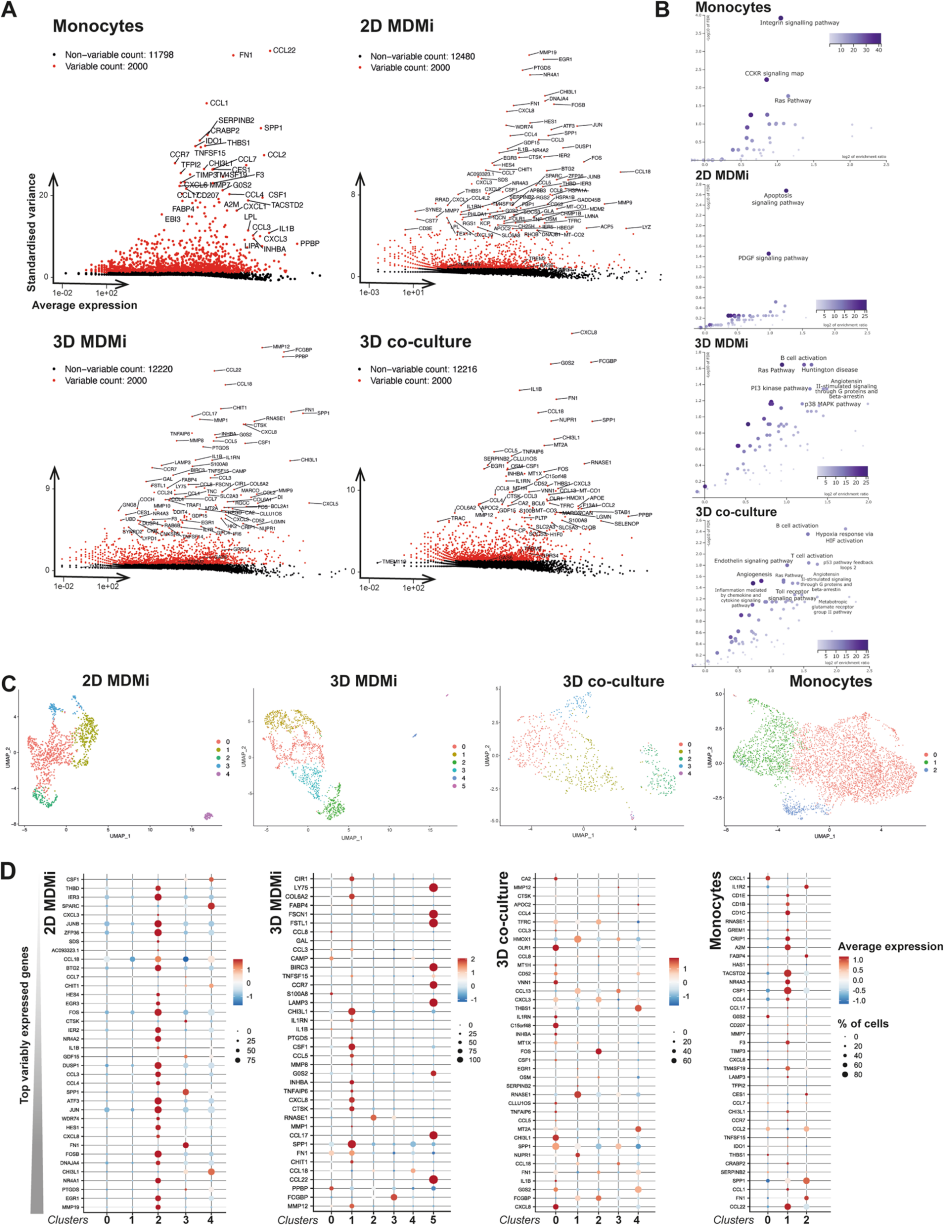
MDMi organize in subpopulations that exhibit the highest pathway enrichment in 3D co-culture. **A** Volcano plot representation of the top 100 most differentially expressed genes in monocytes and MDMi (2D, 3D, and 3D co-culture). **B** Pathway enrichment analysis of monocytes and MDMi (2D, 3D, and 3D co-culture) shows different biological processes across all models, with the greatest diversity in biological functions observed in 3D co-culture. The statistical test used was the hypergeometric test, and the correction methods for multiple testing were Benjamini–Hochberg and FDR. Significance level: *P*-value threshold of 0.05. (Detailed analysis pipeline can be found in Methods). **C** UMAP plots of the monocyte and MDMi single-cell transcriptomes identified three clusters in monocytes, five clusters in 2D MDMi and 3D co-culture, and six clusters in 3D MDMi. Total number of cells analyzed were 9521 for monocytes; 5608 for 2D MDMi; 5667 for 3D MDMi and 4543 for 3D co-culture. **D** Top 38 variable genes expressed in MDMi (2D, 3D, and 3D co-culture), with the most variable genes listed at the bottom and the most constant genes at the top of the list. Each row represents the level of expression of a selected key gene. The color of the dot represents the average expression z-score of the cells within the given cluster, while the size of the dot represents the % of cells within that cluster

Young healthy control participants were recruited at QIMR Berghofer Medical Research Institute (QIMRB-MRI), Queensland, Australia. Alzheimer’s disease (AD) and Healthy control (HC) participants were recruited through the *Prospective Imaging Studying of Aging: Genes, Brain and Behaviour study* (PISA) at QIMRB-MRI [31]. The AD cohort included patients with different levels of brain Aβ deposition and disease severity (Table 1). Samples from young donors (Table 1) were used for the initial optimization and characterization of MDMi in 2D, 3D and 3D co-cultures (Figs. 1, 2; Additional file 1: Fig. S1, S2). Samples from HC and AD donors were used to study disease-associated features in MDMi models and drug testing. These samples were randomly selected, with matching sex, age, and *apolipoprotein E* (*APOE*) status for each assay (Tables 1, 2). *APOE* genotyping was performed in the Sample Processing Facility at QIMRB-MRI, as previously described [31]. The number of biological replicates (*i.e.*, donors), as indicated in figure legends, varied in each assay due to the limited proliferative capacity of MDMi, and the quantity of blood samples available from each donor. Further, repeated longitudinal sampling of peripheral blood from patients was not within the scope of this study.

**Table 1:** Summary of donor information

Study cohorts		Young healthy control	Healthy control (HC)	Alzheimer’s disease (AD)
N° of participants		*n* = 5	*n* = 16	*n* = 16
Sex of participants	Females (%)	40% (2/5)	50% (8/16)	43.8% (7/16)
	Males (%)	60% (3/5)	50% (8/16)	56.3% (9/16)
Age of participants (mean ± SD)		34.2 ± 9.6	67.7 ± 2.8	67.8 ± 6.6
*APOE* genotype	E3/E3 (%)	*N/A*	31.3% (5/16)	18.8% (3/16)
	E3/E4 (%)		56.3% (9/16)	62.5% (10/16)
	E4/E4 (%)		12.5% (2/16)	18.8% (3/16)
Brain Aβ burden	Low (%)	*N/A*	*N/A*	6.3 (1/16)
	High (%)			37.5 (6/16)
	Very high (%)			56.25 (9/16)
Clinical consensus	AD (%)	*N/A*	*N/A*	68.8 (11/16)
	Mild cognitive impairment (MCI) (%)			6.3 (1/16)
	MCI with AD pathology (%)			25 (4/16)

*SD*  standard deviation, *APOE* apolipoprotein E

**Table 2:** Summary of donor information from cohorts used in drug studies

Study cohorts used in drug studies		Healthy control (HC)	Alzheimer’s disease (AD)
N° of participants		*n* = 5	*n* = 8
Sex of participants	Females (%)	60% (3/5)	75% (6/8)
	Males (%)	40% (2/5)	25% (2/8)
Age of participants (mean ± SD)		69.4 ± 2.5	68.5 ± 6.4
*APOE* genotype	E3/E3 (%)	20% (1/5)	0% (0/8)
	E3/E4 (%)	60% (3/5)	75% (6/8)
	E4/E4 (%)	20% (1/5)	25% (2/8)

### Isolation of peripheral blood mononuclear cells (PBMCs)

Peripheral venous blood samples were collected in ethylenediaminetetraacetic acid (EDTA) tubes (Becton-Dickson, NJ, USA). PBMC separation was performed within 2 h of blood withdrawal using SepMate™ tubes (StemCell Technologies, BC, Canada) as per manufacturer’s instructions. PBMCs were washed twice with PBS containing 1 mM EDTA and subsequently frozen in medium containing 10% dimethyl sulphoxide (DMSO) (Merck KGaA, Hesse, Germany) and 90% fetal bovine serum (FBS) (ThermoFisher Scientific, CA, USA) (v/v).

### Establishment of 2D and 3D MDMi cultures

MDMi in 2D were generated as described previously [23, 32]. Briefly, PBMCs were seeded onto Matrigel-coated plates (Corning, NY, USA). After 24 h incubation at 37 °C, 5% CO_2_, a cell population enriched in monocytes remained adhered to the culture vessel. Monocytes were then cultured in serum-free RPMI-1640 GlutaMAX medium (Life Technologies, Grand Island, NY, USA) supplemented with 0.1 μg/ml of interleukin (IL)-34 (IL-34) (Lonza, Basel-Stadt, Switzerland), 0.01 μg/ml of granulocyte–macrophage colony-stimulating factor (GM-CSF) (Lonza) and 1% (v/v) penicillin/streptomycin (Life Technologies) for 14 days. To induce MDMi differentiation in 3D, monocytes were resuspended in Matrigel diluted with ice-cold culture medium at a 1:3 ratio. Matrigel–cell mixtures were seeded in 96-well plates with medium containing 0.1 μg/ml IL-34 and 0.01 μg/ml GM-CSF. 3D MDMi were collected or used for downstream assays after 35 days in culture.

### Establishment of 2D and 3D human neural progenitor cell (NPC) cultures

The human ReNcell VM immortalized neural progenitor cell line (EMD Millipore, Billerica, MA, USA) was cultured as per manufacturer’s instructions, with some modifications. Briefly, cells were plated onto Matrigel-coated plates for 2D cultures or mixed with a 1:3 Matrigel dilution to initiate the 3D cultures. ReNcell VM cultures were maintained in DMEM/F12 GlutaMAX medium (Life Technologies, Grand Island, NY, USA) containing 2% (v/v) B27 supplement, 20 μg/ml epithelial growth factor (EGF) (Sigma-Aldrich, MO, USA), 20 μg/ml fibroblast growth factor 2 (FGF-2) (Lonza, Basel-Stadt, Switzerland) and 1% (v/v) penicillin/streptomycin. Both 2D and 3D cultures were spontaneously differentiated by withdrawing growth factors from the maintenance medium (ReN base medium). All cells used were in passages 7–10 to ensure consistent spontaneous neuro-glial differentiation across independent experiments.

### Establishment of 3D co-cultures (MDMi and ReNcell VM)

ReNcell VM were plated in 3D as described above and cultured for 1 day in ReN base medium to induce spontaneous differentiation. Monocytes were embedded in a 1:3 Matrigel dilution and seeded with 3D ReNcell VM cultures at 1:2.5 to 1:5 monocyte to ReNcell VM ratios. 3D co-cultures were maintained in 50% (v/v) ReN base medium and MDMi culture medium for 35 days.

### Monocyte and MDMi isolation for single-cell RNA sequencing (scRNAseq)

*Monocyte isolation*. PBMCs were thawed and plated in RPMI-1640 GlutaMAX media supplemented with 10% heat-inactivated FBS and 1% penicillin/streptomycin. The next day, cell supernatant containing the leukocyte fraction was discarded and the culture was rinsed thrice with RPMI-1640 GlutaMAX containing 1% penicillin/streptomycin. The remaining adherent monocytes were flushed several times using a 1 ml pipette. Monocyte fraction was then transferred into a fresh collection tube and spun at 300 *g* for 5 min. Cell supernatant was discarded and cells resuspended with 10 ml of PBS and spun at 300 g for 5 min to remove residual FBS.

*2D MDMi isolation*. 14-day MDMi were cultured and lifted using a non-enzymatic detachment method. Briefly, media was discarded, rinsed thrice with ice-cold PBS and replaced with ice-cold cell recovery solution (Corning, #354253). Cultures were incubated at 4 °C for 30 min, with frequent pipetting until all cells were detached. After detachment, cells were washed thrice with PBS supplemented with 5 mM EDTA and centrifuged at 400 *g* for 5 min.

*3D MDMi and 3D co-culture isolation*. To isolate 30-day 3D mono-cultures and co-cultures, supernatant was first removed and cells were rinsed thrice with ice-cold PBS. Ice-cold cell recovery solution was added and cultures were incubated at 4 °C for 45 min with frequent pipetting until Matrigel was dissolved into solution. Cells were rinsed thrice with PBS supplemented with 5 mM EDTA and spun at 400* g* for 5 min to remove residual Matrigel.

### Monocyte and MDMi sorting and fixation for scRNAseq

After harvesting, a cell count was performed and adjusted accordingly (1–10 × 10^6^ cells per 100 μL of PBS containing Zombie Aqua fixable (BioLegend, 423101)), and incubated in the dark at RT for 15 min. After staining, cells were washed twice with PBS containing 0.05% BSA (0.22 μm filtered) and the cell suspension was incubated in cold PBS containing 0.05% BSA and anti-CD11b fluorescein isothiocyanate (FITC) (BioLegend, 101206) for 30 min. After incubation, the cell suspension was washed twice in cold PBS containing 0.05% BSA, filtered through a 40-µm filter and the cells were sorted on a BD FACS Aria II. Cells were sorted into a FACs tube containing PBS and 0.05% BSA, spun down and fixed according to the guide CG000478 for the fixation of cells for Chromium fixed RNA profiling workflow. Briefly, sorted cells were spun at 500 g for 5 min at 4 °C and 1 ml of fixation buffer was added and incubated at 4 °C overnight.

The following day, fixed cells were spun at 850 *g* for 5 min at RT, supernatant was removed and replaced with 1 ml of cold quenching buffer. A cell count via trypan blue exclusion was performed using a Countess II Automated counter (Thermo Fisher Scientific). Fixed cells were then prepared for long-term storage by supplementing with enhancer and glycerol solution and kept at − 80 °C until all samples were ready for library construction.

To minimize the background staining of cells, dyes and antibodies were titrated for optimal performance. All sorting was performed using 100 µm nozzle. This protocol yielded cells that are CD11b-positive (thereby excluding leukocytes and ReNcell VM) (Additional file 1: Fig. S6). Longitudinal blood samples were collected from a single healthy male control donor who was < 40 years of age, did not have dementia, and had no family history of AD. The blood collections were performed twice per month over a 4-month period, resulting in multiple time points. Blood samples from each time point were used for each culture model to serve as technical replicates, to reduce potential biases and improve reliability. Between 230,000 and 400,000 live cells were collected after FACS.

### 10X Genomics Chromium Next GEM single cell fixed RNA library construction and sequencing

Fixed samples for scRNAseq were then processed using the Chromium Next GEM single cell fixed RNA kit (10X Genomics, #PN-1000414 and #PN-1000474) following the standard manufacturer’s instructions. Briefly, fixed single cell suspensions were thawed at RT and processed according to guide CG000477. The cells were loaded onto the Chromium Single Cell Chip Q (10X Genomics, #PN-1000418) to target 10,000 cells. GEM generation and barcoding, and library construction were performed according to the 10X Genomics Chromium User Guide.

Libraries were quantified on the Agilent BioAnalyzer 2100 using the High Sensitivity DNA Kit (Agilent, #5067-4626). Libraries were pooled in equimolar ratios, and the pool was quantified by qPCR using the KAPA Library Quantification Kit via the Illumina/Universal (KAPA Biosystems, KK4824) in combination with the Life Technologies Viia 7 Real-Time PCR instrument (Thermo Fisher Scientific).

Denatured libraries were loaded onto an Illumina NextSeq-2000 and sequenced using a P2 100 cycle flow cell as follows: 28bp (Read1), 8bp (i7 index), 111bp (Read2). Read1 supplies the cell barcode and UMI, i7 the sample index, and Read2 the 3’ sequence of the transcript. Sequencing was performed at the Institute for Molecular Bioscience Sequencing Facility (IMB, University of Queensland). FASTQ files were generated using the CellRanger (7.1.0) pipeline.

### scRNAseq analysis

scRNAseq data were analyzed using the Seurat package (version 4.3.1) in R (version 4.2.3). Raw sequencing reads were processed and aligned to the reference genome (GRCh38-2020-A) using CellRanger (version 7.1.0). The resulting count matrix was imported into R using the Seurat function Read10X().

Quality control was performed by removing cells with low read depth or high mitochondrial gene content using the Seurat function FilterCells(). Cells with more than 2.5% mitochondrial genes were filtered out as well as genes expressed in fewer than three cells or with low expression levels were removed using the Seurat function FilterGenes(). Data normalization was performed using the Seurat function NormalizeData(), and data scaling was performed using the Seurat function ScaleData().

Principal component analysis (PCA) was performed using the Seurat function RunPCA() to identify the principal components that captured the most variation in the dataset. The top 15 principal components (PCs) were used for dimensionality reduction using the Seurat function FindNeighbors() and visualization using the Seurat function RunUMAP(). The top 15 PCs were selected based on the elbow plot that revealed that this first 15 PCs explained more than 95% of the variation in the dataset.

### Clustering and pathway analysis

Cell clustering was performed using the Seurat function FindClusters() with a resolution parameter of 0.5 to identify distinct cell populations. Differential gene expression analysis was performed using the Seurat function FindMarkers() to identify genes that were differentially expressed between cell populations.

To identify cell type and cluster specific markers, we used the Seurat function FindAllMarkers() with the parameters logfc.threshold = 0.25 and min.pct = 0.25. Gene set enrichment analysis was performed using the Seurat function FindMarkers() with the parameters test.use = “wilcox” and logfc.threshold = 0.25 to identify enriched gene sets in each cell population.

The resulting lists of markers were used to perform pathway analysis using the Panther pathway analysis module of the WebGestalt platform. We selected the Panther database as our pathway database and uploaded the list of differentially expressed genes as the input gene list. A pre-defined background gene list from the database was selected as the comparison group. The statistical test used was the hypergeometric test, and the correction method for multiple testing was the Benjamini–Hochberg and false discovery rate (FDR) methods. The significance level was set at a *P*-value threshold of 0.05. The enriched Panther pathways were identified based on the analysis results. The biological significance of the identified pathways was interpreted by examining the gene content and biological functions of the pathways. Functional enrichment analysis was performed by using the ‘clusterProfiler’ package in R to further investigate the biological processes and molecular functions associated with the identified pathways. Pathway visualization tools, such as the ‘pathview’ package in R, were used to visualize the differentially expressed genes within the enriched pathways using the volcano plot showing the significantly enriched pathways.

### Comparison analysis with human microglia datasets

We conducted a Spearman correlation analysis of each of our Monocytes and MDMi (2D, 3D and 3D co-culture) with Olah et al., [33] and Svoboda et al.*,* [34] datasets based on their pseudo-bulk average expression. To this end, we first filtered out low-quality cells based on the number of genes detected and the proportion of mitochondrial genes was set to less than 2.5% as described previously in the methods above. We then obtained the pseudo-bulk expression matrices using the Seurat R package function AverageExpression() which performs average expression of each gene across all cells in the dataset. Thereafter, we obtained the individual pseudo-bulk expression matrices for Monocytes, 2D MDMi, 3D MDMi, 3D co-culture, Olah et al., and Svoboda et al., containing 13,798, 14,480, 14,220, 14,216, 20,063 and 18,983 genes, respectively.

We next performed normalization and batch correction of the pseudo-bulk expression matrices using the Seurat function NormalizeData(). Since the Spearman correlation analysis can only be performed between genes that are common between the two datasets and if there are genes that are unique to one dataset or if there are missing values for some genes, then those genes will not be included in the correlation analysis. Therefore, the correlation analysis of Monocytes, 2D MDMi, 3D MDMi, 3D co-culture with Olah et al., and Svoboda et al.*,* datasets was based on 13,798, 14,480, 14,220 and 14,216 genes, respectively.

We then computed the Spearman’s rank correlation coefficient (ρ) between the average expression values of each gene that was common in both datasets using the cor.test function using the Spearman correlation method in R (version 4.2.3). We then tested the statistical significance of the correlation coefficient using a two-tailed *t*-test with degrees of freedom (n-2), where n is the number of genes. Finally, we adjusted the *P*-values for multiple testing using the Benjamini–Hochberg method to control the FDR and generated a correlation matrix. A scatter plot was used to visualize the correlation between each of our Monocytes, 2D MDMi, 3D MDMi and 3D co-culture datasets with that of Olah et al. and Svoboda et al.

### Immunocytochemistry of 2D and 3D cultures

Immunofluorescence staining of 2D and 3D cultures was performed as described previously [23, 35] with some modifications. 2D cultures were fixed in 4% paraformaldehyde (PFA) or ice-cold methanol (the latter only used for anti-GFAP antibody) for 15 min and blocked at RT with 5% bovine serum albumin (BSA) (Sigma-Aldrich, MO, USA) in PBS. Primary antibodies [TREM 2 (1:500; Abcam, # ab201621), P2RY12 (1:200; Alomone Labs, #APR-20), TMEM119 (1:400; Abcam, # ab185333), IBA1 (1:500; Wako, #019-19741), Nestin (1:200; Abcam, #ab22035), GFAP (1:2000; Abcam, #ab4674), GalC (1:50; Santa Cruz, #sc-518055), Doublecortin (DCX) (1:200; Abcam, ab18723) and βIII-tubulin (TUBB3) (1:500; BioLegend, #801202)] were diluted in blocking solution and incubated overnight at 4 °C. Cells were then washed three times with 0.1% Triton-X 100 in PBS and incubated with secondary antibodies [1:250 Alexa Fluor 488 (#A-11034) / 594 (#A-21203) / 647 (#A-21244) (ThermoFisher Scientific, CA, USA)] for 2 h at RT in the dark and counterstained with a nuclear dye (Hoechst 33342, 1 μg/ml). 3D cultures were fixed with 4% PFA overnight at RT, permeabilized for 30 min with 0.3% Triton-X 100 in PBS, and blocked overnight at 4 °C with 2% BSA (Sigma-Aldrich, MO, USA) in PBS. Primary antibody solutions were incubated for 24 h at 4 °C. Secondary antibody solutions were incubated for 5 h at 4 °C in the dark. Cultures were then washed five times (10 min each) with 0.1% Triton-X 100 in PBS and counterstained with Hoechst 33342. Images were captured using a confocal laser scanning microscope (LSM-780, Carl Zeiss) at 20× and 40× magnification and processed using the Zeiss ZEN software.

### RNA extraction and quantitative real-time PCR (qRT-PCR)

RNA and cDNA were prepared as previously described [36]. Total RNA was extracted using a Direct-zol RNA Miniprep kit (Integrated Sciences, Australia) as per manufacturer’s protocol. Conversion to cDNA was carried out using a SensiFAST™ cDNA synthesis kit (Bioline, London, UK). For qRT-PCR, cDNA was diluted 1:10 to generate working solutions and combined with SensiFAST™ SYBR^®^ Lo-ROX master mix and gene-specific primers. qRT-PCR runs were performed as triplicate on Applied Biosystems ViiA 7 (ThermoFisher Scientific, CA, USA). Endogenous control 18S was used as a housekeeping gene for normalization. Relative gene expression levels were calculated using the ΔΔCt method. Human primer sequences used are listed in Additional file 6: Table S1.

### Multiplex bead-based immunoassay

The LEGENDplex™ Human Inflammation (#740809) and Growth Factor (#740180) kits (BioLegend, CA, USA) were used to detect cytokines and growth factors in conditioned media. The assay was performed as per manufacturer’s instructions. Briefly, conditioned media were incubated with a cocktail of antibody-conjugated capture beads. Then biotinylated detection antibodies were added followed by streptavidin–phycoerythrin (SA-PE). The amount of analytes of interest in the samples was calculated as a proportion of the fluorescent signal intensity provided by capture bead-analyte-detection antibody-SA-PE sandwiches. Signals were acquired on a BD LSRFortessa 5 (BD Biosciences, CA, USA) using FACSDiva software, and analyzed using Qognit, a cloud-based LEGENDplex™ software (BioLegend, CA, USA). Concentrations (pg/ml) were normalized to total amount of protein in the cultures.

### Morphology analysis

Quantification of morphological parameters of MDMi was performed by adapting a previous method [37]. Phase contrast images in 2D cultures were acquired using a spinning disc confocal microscope with a 20X objective of 0.4 numeric aperture. For 3D cultures, we used an Olympus CKX41 inverted microscope at 20X magnification with a 5.1MP CMOS digital camera. Subsequently, these images were processed in FIJI software (National Institutes of Health, Maryland, USA) using a macro script that applied a threshold, followed by processing functions “despeckle”, “close” and “remove outliers” that generated a binary image. Binary images were then run on the AnalyseSkeleton (2D/3D) plugin, which resulted in skeletonized images. The “results and branch information” outputs from the plugin contained data on branch length, branch number and triple and quadruple junctions number. Binary images were also analyzed using the *Analyze particles* function in FIJI. This calculated the “solidity” or “ramification index” value, which results from dividing the area of MDMi by its convex area (i.e., area of the smallest polygon drawn around the cell). More ramified cells have a bigger convex area and thus a smaller ramification index (< 1). Mean single cell values for each parameter were calculated. The total number of MDMi analyzed per donor was 100 in 2D and 20 in 3D. *N* numbers are specified in the corresponding figure legends and represent biological replicates (*i.e*., donors).

### Cell contacts analysis in 3D co-cultures

Confocal Z-stack images of 3D co-cultures acquired with a 20X objective were rendered in 3D using the Imaris software (Bitplane, Belfast, UK) and analyzed with the Surface-Surface contact area extension module. During image acquisition, the Z-interval was set at “Optimal” so that the number of acquired slices was suitable for the given stack size, objective lens, and pinhole diameter. Following surface modeling using the Surface function in Imaris, the Surface-Surface contact area extension module was applied to measure the areas in contact between ReNcell VM and MDMi as well as the number of contacts established. Both parameters were then normalized to the total number of MDMi in the image. A total of 200 MDMi in co-culture were analyzed for each donor in the HC and AD cohorts.

### Preparation of amyloid-β (Aβ) aggregates

FITC-conjugated Aβ peptides 1-42 (FITC-Aβ1-42) (Bachem, M2585, CH) were dissolved in DMSO (Merck KGaA, Darmstadt, Germany) to a concentration of 500 μM and stored at − 80 °C. FITC-Aβ1-42 were incubated for 24 h at 37 °C in the 3D cultures prior to imaging to allow for the fibrillization of the peptides and formation of Aβ aggregates.

### Aβ aggregates exposure and surveillance analysis

FITC-Aβ1-42 peptides were added at 5 μM to MDMi 3D mono- and co-cultures at day 35 of differentiation. After 24 h, cultures were imaged on an EVOS FL Auto 2 (ThermoFisher Scientific, CA, USA). Scans were set to image multiple z-stack planes every 12 h for 7 days using a 10X objective. At least 3 fields of view were scanned per well. MDMi located within an area of 90,000 μm^2^ containing one or more Aβ aggregate were tracked using the Manual tracking plugin in FIJI. Migrated distance and speed of tracked cells over 7 days of live-imaging were calculated and normalized to the number of MDMi in the analyzed areas. Between 100 and 200 MDMi per individual were tracked in both HC and AD cohorts. *N* numbers are specified in the corresponding figure legends and represent biological replicates (i.e., donors).

### Drug treatments

The FDA-approved drugs dasatinib (#CDS023389, Sigma-Aldrich, MO, USA) and spiperone (#S7395, Sigma-Aldrich, MO, USA) were reconstituted in DMSO (Merck KGaA, Darmstadt, Germany) at a working concentration of 100 nM and 1 μM, respectively. Drug treatment duration was 24 h and was performed in 2D cultures at day 14 of differentiation, and 3D cultures at day 35 of differentiation. MDMi in both 2D and 3D models were simultaneously established using monocytes collected from the same blood sample obtained from each donor. Donors were matched for sex, age and *APOE* status (Table 2). Consistent with the drug solvent, a vehicle control treatment consisting of DMSO at a concentration below 0.1% v/v for 24 h served as a normalization control. Drug responses are presented as fold change relative to vehicle-treated cultures (drug/vehicle).

### Statistical analysis

All statistical analyses were performed using GraphPad Prism software version 8 (Graphpad Software, CA, USA). Comparisons between two groups were analyzed with two-tailed Student’s *t*-test or Mann–Whitney U, when normality assumptions were not met. Assumption of normality was determined using the Shapiro–Wilk normality test. Comparisons between three or more groups were analyzed by one- or two-way analysis of variance (ANOVA) followed by post hoc tests (Dunnett’s multiple comparison test for one-way ANOVA analyses; Tukey’s or Šídák’s post hoc test for two-way ANOVA analyses). These tests have been specified in the corresponding figure legends. Unless specified otherwise, data points and *n* represent biological replicates (independent donors). For correlation analysis with *APOE* genotype, a Spearman rank correlation test was performed. Outliers were calculated using the Grubbs’ test Extreme Studentized Deviate (ESD) method provided by GraphPad software (https://www.graphpad.com/quickcalcs/Grubbs1.cfm). Data are presented as mean ± SEM or mean ± SD and *P* ≤ 0.05 was considered significant. Statistical significance was determined as *P < 0.05, **P < 0.01, ***P < 0.001, ****P < 0.0001, as detailed in figure legends.

## Results

### Single-cell gene expression profiling of MDMi shows a separate signature from monocytes and a closer resemblance of the 3D models to bona fide human microglia

To assess to influence of the culture environment on the acquisition of a microglia-like signature in MDMi, we differentiated monocytes into MDMi in 2D and 3D mono-cultures, and in 3D co-culture with neuro-glial cells (Fig. 1A). Positive immunostaining for the microglia-enriched markers Tmem119, P2ry12, Trem2 and Iba1 (Fig. 1A) confirmed the acquisition of a microglia-like identity in MDMi.

In 3D mono-cultures, Matrigel embedding resulted in thicker cultures (6.2-fold increase) with extended cell survival (2.5-fold increase) compared to 2D (Additional file 1: Fig. S1A; Additional file 8: Movie S1, Additional file 9: Movie S2). Morphologically, the 3D model induced a significantly more ramified pattern (Additional file 1: Fig. S1B) in MDMi that may result from the larger surface area for growth and differentiation provided by the 3D Matrigel scaffold.

In the 3D co-culture model, we mimicked the neuro-glial cues of the brain microenvironment by using the immortalized human neural progenitor cell (NPC) line ReNcell VM [38]. The spontaneous differentiation of ReNcell VM in 2D over 30 days led to a mixed population of radial glia, astrocytes, oligodendrocyte progenitor cells (OPCs) and neurons (Additional file 1: Fig. S2A, B). ReNcell VM differentiation in a 3D Matrigel-based culture showed decreased proliferative capacity along with increased mature astrocyte and neuronal marker expression compared to 2D (Additional file 1: Fig. S2C, D). In addition, we observed that 2D ReNcell VM cultures did not support MDMi differentiation in co-culture given that monocytes retained a round morphology in long-term 2D co-cultures (Additional file 1: Fig. S3A), thereby suggesting that a 3D Matrigel scaffold is necessary for co-culturing MDMi and ReNcell VM. In 3D co-culture the inflammatory function of MDMi was intact, as indicated by the increased secretion of classical pro-inflammatory cytokines (IL-6, IL-1β and IL-18) following the exposure to FITC-Aβ peptides (Additional file 1: Fig. S3B).

To characterize MDMi in the different model systems at a deeper level, we performed single-cell RNA sequencing (scRNAseq). For scRNAseq analysis, we generated MDMi cultures using monocytes derived from a young healthy male donor (Table 1). Single cell suspensions of monocytes and MDMi were obtained by non-enzymatic cell recovery, and CD11b-positive cells were sorted and fixed using the 10X Genomics Chromium fixed RNA profiling protocol (Fig. 1B). A CD11b-positive selection was used to specifically isolate myeloid cells (monocytes and MDMi) and exclude other cell types such as leukocytes and ReNcell VM (Additional file 1: Fig. S4). Fixed cells were preserved at – 80 °C until all samples were ready for subsequent library preparation. Following quality control (Additional file 1: Fig. S5), a total of 9521 monocytes; 5608 MDMi in 2D; 5667 MDMi in 3D and 4543 MDMi in 3D co-culture were analyzed.

We conducted unsupervised clustering and uniform manifold approximation and projection (UMAP) dimensional reduction techniques to investigate the differences in single-cell transcriptomic profiles between MDMi and monocytes. The UMAP plot clearly indicated a separation between monocytes and MDMi in 2D, 3D and 3D co-culture models (Fig. 1C). We observed that the expression of genes enriched in monocytes (*CCL22*) and microglia (*C1QA*, *TREM2*, *PROS1*, *GPR34*, *TMEM119*, *GAS6*) was highly variable across models (Fig. 1D; Additional file 1: Fig. S6A). Indeed, the expression of microglia-enriched genes within the population of MDMi cells in each model system displayed heterogeneity, with less than 100% of the cells expressing these genes. This confirms the presence of various MDMi subpopulations with unique transcriptional features, indicating a diversity of cellular states resembling brain microglia. Overlaying the expression of selected genes in the UMAP plot demonstrated that MDMi models exhibited a decrease in monocyte markers (*CCR2, CCL22*), retention of myeloid markers (*CD14*), and an upregulation of *bona fide* microglia markers (*AIF1, C1QA, GPR34, PROS1, GAS6, CST3, TMEM119*, *TREM2, MERTK, P2RY12, CD74*) compared to monocytes (Fig. 1E, F; Additional file 1: Fig. S6B). Notably, microglial genes that have been implicated in neurodegeneration, including *APOE*, *MS4A4A*, *C3*, *A2M*, *SPP1* and *FCGBP*, showed variable expression levels and distributions across MDMi models (Fig. 1G).

To investigate the shared characteristics among MDMi cultures and determine the conservation of the monocytic transcriptomic signature, we partitioned the clusters according to model system and depicted them using a UMAP plot (Fig. 1H). A comprehensive list of significantly differentially expressed genes (*P* < 0.05) in each individual cluster can be found in Additional file 2: Dataset S1. We observed that cluster 8, which was defined by biological pathways associated with inflammation, integrin signaling and sexual steroid biosynthesis, was common in monocytes and all MDMi models (Fig. 1I; Additional file 1: Fig. S7). In contrast, clusters 2, 3, 4, 5 and 9 were commonly found in MDMi models but not in monocytes. These clusters were enriched in p53, PDGF, CCKR and neurotransmitter signaling pathways (Additional file 1: Fig. S7). Cluster 6 (associated with cytoskeletal regulation and plasminogen activation) was exclusive to 2D MDMi, whereas monocytes shared clusters 7 and 10 (associated with toll receptor, p53 signaling and T cell activation) with 3D MDMi, and cluster 0 (associated with toll receptor and CCKR signaling) with 3D co-culture (Fig. 1I; Additional file 1: Fig. S7).

We then assessed the similarity between MDMi and *bona fide* human microglia by comparing the scRNAseq expression profiles of these cells against published datasets of post-mortem microglia derived from healthy individuals (Olah et al. 2020) [33] and hiPSC-derived microglial cells differentiated in vitro in 2D over 60 days (Svoboda et al. 2019) [34]. Pseudo-bulk analysis was used to calculate the average expression of common genes, and the datasets were compared using correlation scatter dot plots. The following number of genes were included in the correlation tests: 13,798 for monocytes; 14,480 for 2D; 14,220 for 3D and 14,216 for 3D co-culture. In 3D co-culture MDMi exhibited the strongest correlation with both Olah et al. (R = 0.78) and Svoboda et al. (R = 0.81) datasets, while 2D MDMi showed a weaker correlation (R = 0.55 with Olah et al., R = 0.63 with Svoboda et al.) (Fig. 1J, K). Importantly, monocytes showed little resemblance with both these well-established microglia models (R = 0.42 with Olah et al., R = 0.27 with Svoboda et al.) (Fig. 1J, K). Correlation of the pseudo-bulk expression profile of a subset of 798 core microglial genes [13, 33, 39] confirmed that 3D co-culture MDMi are more similar to Olah et al. (R = 0.70) and Svoboda et al. (R = 0.57) than the other culture systems (Additional file 1: Fig. S8; Additional file 3: Dataset S2). However, we noted that *SALL1*, a driver of microglial identity [40], was not expressed in any of our MDMi models nor in Svoboda et al., suggesting a limitation of these in vitro models to recapitulate all aspects of microglial biology.

Our findings highlight that MDMi in 3D co-culture more closely mimic human microglia relative to mono-culture models, and offer advantages over 2D hiPSC-derived mono-cultures by capturing the microglial signature in a more physiologically relevant culture environment and a shorter timeframe (30 days).

### Microglial functional heterogeneity is best captured in 3D-cultured MDMi

Our results demonstrate that 3D modeling significantly improves unique microglial characteristics in MDMi, including a more ramified morphology and a transcriptomic signature that more closely resembles well-established in vitro models of human microglia. However, an important aspect of these cells is that in the human brain, they organize in subpopulations that drive disease development and could respond more efficiently to targeted therapeutics [41]. Therefore, it is key that microglial heterogeneity can be captured by human in vitro models.

To investigate if MDMi in 3D models recapitulate the natural heterogeneity of microglial cells better than 2D, we analyzed the single-cell transcriptomes of monocytes and MDMi. A volcano plot representation of the top 100 most differentially expressed genes (DEGs) in monocytes and MDMi in each model system revealed distinct transcriptomic profiles (Fig. 2A), which exhibited enrichment in diverse biological processes (Fig. 2B). Notably, the transcriptional states of monocytes and 2D MDMi demonstrated a narrower range of pathway involvement in comparison to their 3D mono- and co-culture counterparts (Fig. 2B), which suggests that the 3D environment may promote increased functional diversity in MDMi.

Individual UMAP analyses of monocytes and MDMi identified five clusters in 2D and 3D co-culture, six clusters in 3D and only three clusters in monocytes (Fig. 2C). This suggests that MDMi exhibit a greater cellular heterogeneity and more diverse gene expression profiles compared to monocytes. All differentially expressed genes (*P* < 0.05) within each individual cluster for every model system are listed in Additional file 4: Dataset S3. Upon comparing variable gene expression signatures among clusters, we observed the presence of markers enriched in microglia and macrophages [42], including *SPARC*, *CTSK*, *FOS*, *FN1*, *OLR1*, *COL6A2* and *SPP1* (Fig. 2D). Moreover, the differential expression of the homeostatic markers *EGR3* and *CCL4 *[43], which have been associated with pre-active microglia in human brains, suggests varying activation states of the MDMi subpopulations (Fig. 2D). Enrichment analysis of DEGs within the top three clusters in each model system indicated involvement of interferon responses and TNF-α signaling in 2D MDMi; complement, TNF-α and PI3K/AKT/mTOR signaling in 3D MDMi; and complement, hypoxia, and mitotic spindle pathways in 3D co-culture (Additional file 1: Fig. S9).

Collectively, the identification of more transcriptionally and functionally distinct subpopulations of microglia-like cells confirms that MDMi in the 3D models attain a variety of cellular states akin to microglia within the human brain.

### MDMi generated from AD patients exhibit disease-specific phenotypes

**Fig. 3:**
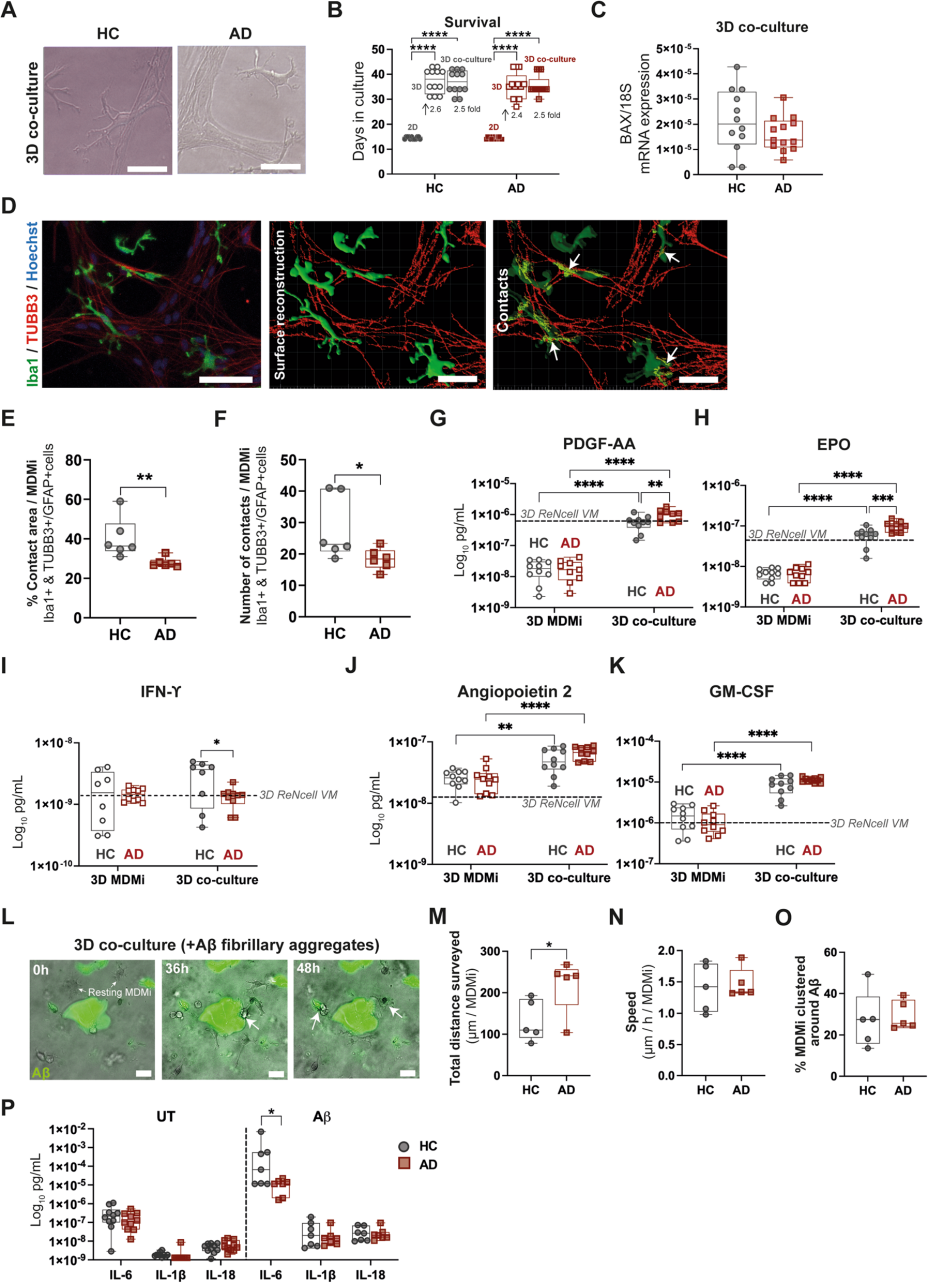
AD-associated phenotypes observed in patient-derived 3D co-cultures. **A** Representative bright field images of HC and AD co-cultures. Scale bars, 100 μm. **B** Survival of HC (*n* = 12) and AD (*n* = 13) MDMi in 2D and 3D mono-cultures, and 3D co-cultures. Arrows and values indicate the fold change increase of the mean survival in 3D and 3D co-cultures versus 2D in each cohort. **C** Gene expression of the pro-apoptotic marker *BAX* in HC (*n* = 12) and AD (*n* = 13) 3D co-cultures. **D** Immunostaining against Iba1 (green) and Tubb3 (red) and 3D surface reconstruction of a 3D co-culture. Areas of contact between Iba1 + (MDMi) and Tubb3 + (ReNcell VM) cells are highlighted in yellow (white arrows). Scale bars, 100 μm. Quantification of **E** contact area and **F** number of contacts between Iba1 + and Tubb3 + /Gfap + cells in HC (*n* = 6) and AD (*n* = 6) 3D co-cultures. Concentration of **G** platelet-derived growth factors AA (PDGF-AA), **H** erythropoietin (EPO), **I** interferon-γ (IFN-γ), **J** angiopoietin 2 and **K** granulocyte–macrophage colony stimulating factor (GM-CSF) secreted by HC (*n* = 8–10) and AD (*n* = 9–10) MDMi in 3D mono-cultures and co-cultures. Baseline secretion by 3D ReNcell VM mono-cultures is represented with a dotted black line. **L** Representative images of 3D co-cultures containing FITC-Aβ aggregates. MDMi exhibit a resting, ramified morphology at 0 h. They progressively become polarized and acquire an activated, round morphology with enlarged soma upon reaching and clustering on the Aβ deposit at 36 h and 48 h (white arrows). Scale bars, 100 μm. See also Additional file 10: Movie S3. **M** Total surveillance distance, **N** speed and **O** clustering around Aβ aggregates of HC (*n* = 5) and AD (*n* = 5) MDMi in 3D co-cultures. **P** Concentration of pro-inflammatory cytokines IL-6, IL-1β and IL-18 secreted by untreated (UT) and Aβ-treated HC (*n* = 10 UT cultures; *n* = 7 Aβ-treated cultures) and AD (*n* = 10 UT cultures; *n* = 7 Aβ-treated cultures) MDMi in 3D co-cultures. Data are presented as mean ± SD. Data points and *n* represent biological replicates (donors). One-way ANOVA with Tukey’s multiple comparison test in **B**; unpaired Student’s *t* test with or without Welch’s correction, two-tailed in **M, P**; Mann–Whitney test, two-tailed in **E**, **F**; two-way ANOVA with Tukey’s multiple comparison test in **G–K**; *P < 0.05, **P < 0.01, ***P < 0.001, ****P < 0.0001

To assess if patient-derived MDMi models capture disease-specific features of AD microglia, we generated 2D and 3D MDMi using monocytes derived from AD patients and healthy control (HC) individuals matched for sex, age and *APOE* genotype (Fig. 3A; Table 1). We first confirmed that the origin of the cells did not affect the survival of MDMi in culture. We observed that both 3D models (mono-culture and co-culture) induced an average 2.5-fold increase in cell survival compared to 2D MDMi irrespective of cohort (Fig. 3B). Consistently, similar expression of the apoptosis marker *BAX* between HC and AD 3D co-cultures (Fig. 3C) suggests that the cell ratio of MDMi and ReNcell VM and the duration of the co-culture were favorable.

We noted stronger disease-associated features in 3D co-culture (Fig. 3) than in the mono-culture (Additional file 1: Fig. S10) models. While MDMi mono-cultures showed no morphological differences between cohorts, a trend for an upregulated expression of disease risk genes (including *CLU*, *TREM2*, *PLCG2* and *PILRB*) was observed in AD 3D MDMi (Additional file 1: Fig. S10).

In 3D co-culture, we observed disease-specific alterations at different levels. Firstly, the 3D rendering and subsequent surface reconstruction of 3D co-culture images revealed a significantly smaller area of contact and a reduced number of contact points between MDMi and ReNcell VM in AD compared to HC 3D co-cultures (Fig. 3D–F). This indicates a possible impairment in the cell-to-cell interactions between MDMi and ReNcell VM in AD 3D co-cultures, and could provide a mechanistic basis for the microglia-driven aberrant synaptic engulfment that occurs in AD [44]. Secondly, comparison of HC and AD MDMi secretory profiles in 3D co-culture showed that AD cells secreted higher levels of platelet-derived growth factor AA (PDGF-AA) and erythropoietin (EPO), and lower levels of interferon-γ (IFN-γ), compared to HC (Fig. 3G–I; Additional file 5: Dataset S4). This indicates that AD MDMi exhibit an altered secretory activity in the 3D co-culture. Moreover, we observed a significant upregulation of PDGF-AA, EPO (Fig. 3G–I) and other neurotrophic factors such as Angiopoietin 2 and the granulocyte–macrophage colony stimulating factor (GM-CSF) (Fig. 3J, K) in 3D co-cultures from both HC and AD cohorts compared to their 3D mono-culture counterparts. Secretion of these factors by 3D ReNcell VM mono-cultures (dotted lines in Fig. 3G–K) changed compared to 3D MDMi mono-cultures, suggesting that the interaction between MDMi and ReNcell VM in the 3D co-cultures has a functional impact on both cell types. Lastly, AD MDMi exhibited altered behaviors in the presence Aβ aggregates in the 3D co-cultures. Specifically, upon adding FITC-Aβ peptides into HC and AD 3D co-cultures and live-imaging for 7 days (Fig. 3L; Additional file 10: Movie S3), we observed that AD MDMi surveyed longer distances around Aβ aggregates compared to HC, with no significant changes in speed and proportion of cells clustering around these aggregates (Fig. 3M–O). Aβ exposure also elicited disease-specific inflammatory responses in the 3D co-cultures, with IL-6 being significantly secreted at lower levels in AD compared to HC (Fig. 3P; Additional file 5: Dataset S4). Of note, none of the observed disease-associated phenotypes showed a significant correlation with *APOE* genotype (Additional file 7: Table S2).

Combined, these results indicate that AD patient-derived MDMi exhibit most disease-related alterations when modeled in 3D co-culture.

### Alzheimer’s disease patient-specific MDMi models may facilitate the translatability of drug pre-clinical screens

**Fig. 4:**
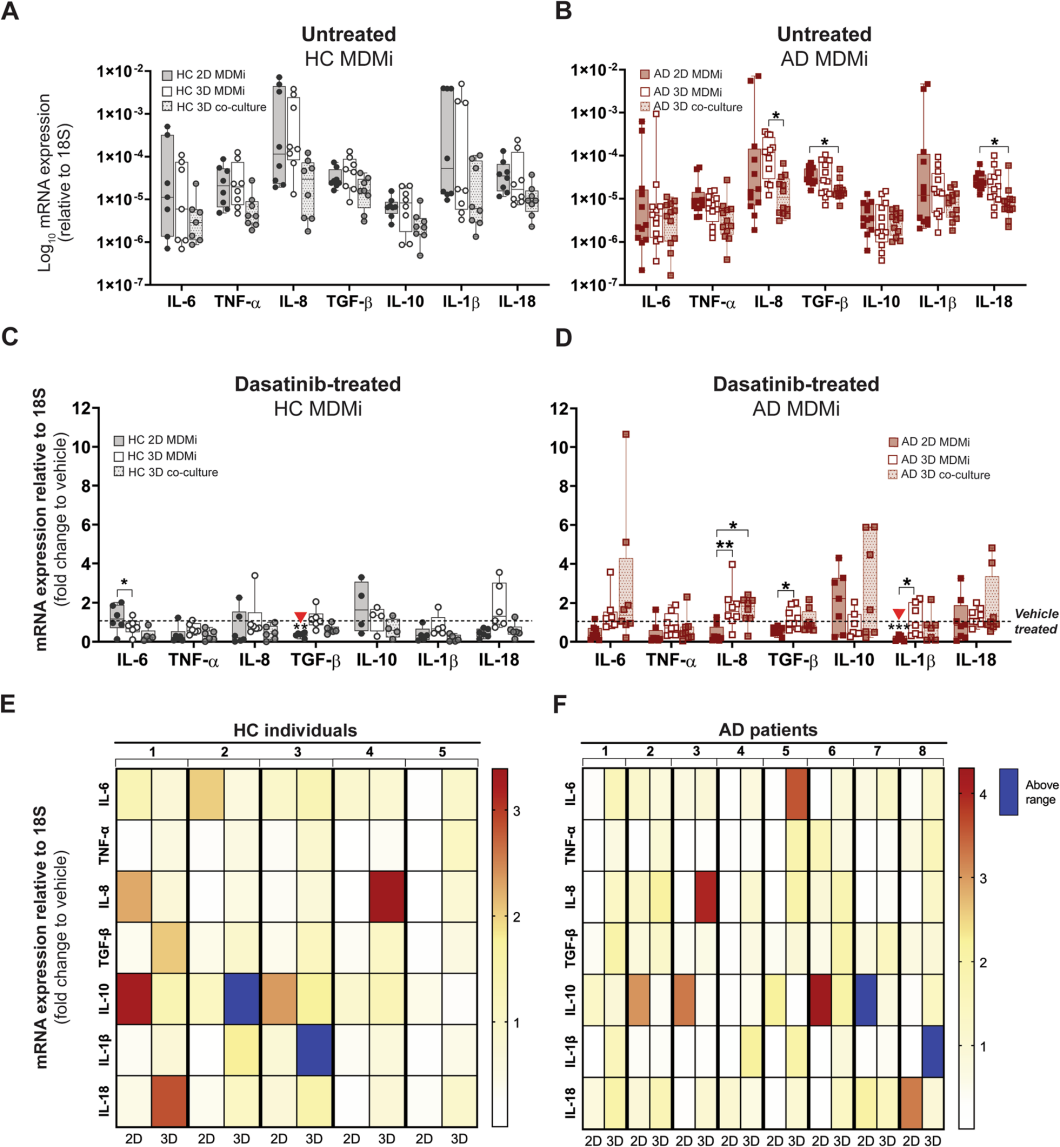
Dasatinib induces patient- and model-specific cytokine responses. **A, B** Log-transformed mRNA expression of the inflammatory cytokines *IL-6*, *TNF-α*, *IL-8*, *TGF-β*, *IL-10*, *IL-1β* and *IL-18 *in untreated HC (*n* = 7–8) and AD (*n* = 12) MDMi (2D, 3D and 3D co-cultures). **C, D** Fold change of cytokine mRNA expression levels following 24 h exposure to 100 nM dasatinib compared to vehicle (DMSO)-treated cultures in HC (*n* = 4–6) and AD (*n* = 7–8) MDMi (2D, 3D and 3D co-cultures). Red arrowheads indicate significance compared to vehicle-treated condition. Dotted black lines represent baseline responses of vehicle-treated cultures. **E, F** Heat maps showing HC (*n* = 5) and AD (*n* = 8) donor-specific changes in mRNA expression from 2D and 3D MDMi mono-cultures. Red-yellow color spectrum represents relative fold change of mRNA expression after dasatinib treatment compared to vehicle. Expression changes falling outside the displayed range are indicated in dark blue. Data are presented as mean ± SD. Data points and *n* represent biological replicates (donors). One-way ANOVA with Dunnett’s multiple comparison test; **P < 0.01, **P < 0.01, ***P < 0.001

Using the right culture model is essential for yielding accurate and clinically translatable outcomes in pre-clinical drug testing assays. To evaluate if responses to drugs were dependent on model system in AD patient-derived MDMi, we trialed two FDA-approved compounds—dasatinib (tyrosine kinase inhibitor) and spiperone (dopamine antagonist). These drugs have been shown to mitigate inflammation in in vitro models of microglia [45, 46] and murine models of AD [47] and have potential as re-purposed drugs for treating neuroinflammation. At baseline (untreated MDMi cultures) minor differences in cytokine gene expression profiles were observed between model systems irrespective of cohort (Fig. 4A, B). Noticeably, in the AD cohort, 3D co-cultures showed a significant downregulation of *IL-8* compared to 3D MDMi, and *TGF-β* and *IL-18* compared to 2D MDMi (Fig. 4B).

Interestingly, these culture-specific differences at baseline changed when HC and AD MDMi (Table 2) were treated with dasatinib (100 nM, 24 h) and spiperone (1 μM, 24 h) (Fig. 4C, D; Additional file 1: Fig. S11). For example, dasatinib changed *IL-6* expression between culture models in the HC cohort (Fig. 4C), while *IL-8*, *TGF-β* and *IL-1β* showed culture-dependent differences in the AD cohort (Fig. 4D). Considering that we performed these drug studies by keeping all experimental conditions constant (*i.e.*, simultaneous differentiation of MDMi in all models using the same batch of monocytes obtained from each respective donor) and only varying the culture environment (*i.e.*, 2D, 3D or 3D co-culture), our data suggest that in vitro drug responses strongly depend on model system. In the context of a drug pre-clinical screen, this observation reinforces the premise that drug efficacy should be validated using an appropriate model system.

To further identify if other variables besides culture environment drive drug responses in MDMi, we used heat maps to compare donor-by-donor cytokine expression (represented as fold change relative to vehicle) between 2D and 3D mono-cultures. For this analysis, we excluded 3D co-cultures to account for drug responses mediated by MDMi alone (mono-cultures) without the influence of ReNcell VM (present in 3D co-cultures). These heat maps showed that dasatinib and spiperone responses not only depend on model system, but also are donor- and cytokine-specific (Fig. 4E, F; Additional file 1: Fig. S11).

This pilot experiment has demonstrated that microglial drug responses are model-dependent and heterogeneous among HC and AD donors. While further studies are required to select the most suitable model system for pre-clinical drug assays, the observed variability underscores the importance to personalize drug development approaches in AD. Lastly, in view of the high variability rates in treatment responses among AD patients [48], patient-derived MDMi models are powerful tools to improve the clinical translatability in AD research by facilitating the design of personalized in vitro drug screens and pre-selecting responders before clinical trials.

## Discussion

Recreating physiologically relevant culture conditions to mimic the interaction of microglia with other brain cell types and the extracellular matrix is crucial for accurately modeling the role of microglia in disease and improving the clinical translation of drug studies. To our knowledge, no study has attempted to culture patient microglia-like cells transdifferentiated from blood monocytes in a culture setting that resembles the human brain. Hence, in an effort to develop more representative in vitro models of human microglia, we used MDMi—a model system of microglia-like cells that has emerged as a potential patient-specific drug screening platform for neurological diseases [49–51]—and developed novel 3D hydrogel-based MDMi platforms as mono-cultures, or in co-culture with human neuro-glial progenitors.

Deep transcriptional phenotyping using scRNAseq revealed differential gene expression signatures of MDMi compared to monocytes, confirming the efficient induction of a microglia-like state in these cells. Importantly, microglia-enriched markers such as *C1QA*, *TREM2*, *PROS1*, *GPR34*, *TMEM119* and *GAS6* were upregulated in MDMi and showed heterogeneous expression levels within the cell subpopulations in each model system. Interestingly, MDMi also exhibited various transcriptional states, which closely mirrors the cellular heterogeneity of microglia in the human brain. In addition, our results showed that MDMi cultured in 3D exhibited enrichment in a more diverse range of biological processes and closer gene expression resemblance to post-mortem and hiPSC-derived microglia [33, 34] than 2D. These results validate the use of MDMi as an accurate in vitro surrogate of human microglia by replicating its transcriptomic signature and heterogeneity. Thus, leveraging MDMi to selectively study specific microglial states from a therapeutic perspective holds promising potential. Moreover, our study revealed that MDMi subpopulations in each model system showed distinct enrichment for genes related to neurodegenerative diseases, including *APOE*, *MS4A4A* and *C3*. This provides a basis for using MDMi for investigating molecular pathways and functional roles related to disease.

Microglia are involved in the pathogenesis of AD and contribute to the clinical heterogeneity observed among patients. 3D MDMi models offer a great opportunity to study AD microglia-like cells in a patient-specific manner. Unlike murine, human immortalized and hiPSC-derived microglia cell lines used in previous studies [52, 53], MDMi are patient-derived and have not been genetically modified, being therefore more physiologically relevant and clinically applicable. In addition, the use of cell samples obtained from living patients allows for the longitudinal modeling of disease progression in AD, a pre-requisite for targeted treatment at various stages of the disease.

Microglia establish physical contacts with neurons through identified molecular mechanisms such as purinergic and mitochondrial signaling [54]. Our results showed reduced cell-to-cell contacts between AD MDMi and ReNcell VM-derived neuro-glial cells compared to HC in 3D co-culture. Alterations in such microglia–neuron interactions could impact the microglial capacity to respond to neuronal damage, providing a potential mechanism underlying neuron degeneration in AD. The interaction between AD MDMi and ReNcell VM revealed altered secretory patterns of specific factors. Dysregulations in these factors have been reported in AD patients. For example, in post-mortem brain samples an imbalanced distribution of PDGF-AA immunopositive cells associated with gliosis and altered protein expression levels of astrocytic EPO receptors were identified [55, 56]. Similarly, in living patients IFN-γ plasma levels were found to vary with disease severity, angiopoietin-2 plasma levels were increased in *APOE*4 carriers and cerebrospinal fluid levels of GM-CSF showed upregulation and significant correlation with tau protein levels [57–59]. Although these alterations have not been directly associated with microglia, AD MDMi provide a suitable in vitro tool to understand the potential role of microglia in these changes.

Microglia have a canonical role in the removal of Aβ aggregates [60]. We showed that AD MDMi migrate longer distances compared to HC. Similarly, elevated migration rates were observed in human immortalized microglia cultured in a 3D tri-culture model with ReNcell VM overexpressing pathogenic Aβ species [52]. However, an AD mouse model with aberrant Aβ production showed decreased microglial migration towards Aβ plaques [61], highlighting important differences between human and murine microglia responses to Aβ. Our findings warrant further investigation of microglial motility as a potentially dysregulated cellular feature in AD brains. Interestingly, we did not observe differences in the number of MDMi that clustered around Aβ aggregates. However, we observed changes in pro-inflammatory cytokine secretion in MDMi from AD compared to HC. This suggests that AD MDMi may have unique disease-specific chemotactic and secretory responses against Aβ. Future studies should investigate how such changes in MDMi impact the phagocytic clearance of Aβ aggregates in the 3D co-cultures. Overall, disease-specific differences exhibited by AD MDMi in 3D co-culture confirm the possibility to model disease in AD patient-specific MDMi using culture models that better recapitulate the brain microenvironment.

Preliminary drug testing demonstrated the utility of the 3D MDMi models as personalized drug screening tools. For instance, differences in MDMi drug responses between 2D and 3D culture conditions reflect the functional impact of MDMi cultured in a more biologically relevant 3D environment. This agrees with studies reporting more similarities in drug-induced cellular responses between 3D cultures and in vivo conditions than compared to 2D cultures [62, 63]. Future investigations should determine whether drug responses from our 3D MDMi models correlate better with responses identified in animal models and clinical data from patients. Moreover, our results revealed donor variability in MDMi drug responses likely derived from the heterogeneity of disease presentation among donors. This observation would support the potential translatability of our 3D platforms to measure individual patient responses in the clinic. The identification of distinct subpopulations of MDMi within the 2D and 3D model systems by scRNAseq profiling could support the hypothesis that MDMi drug responses are mediated by specific cellular subsets. Therefore, understanding the unique roles of these MDMi clusters is crucial in developing more efficient in vitro drug testing approaches of microglia-targeted compounds.

While MDMi offer considerable clinical relevance and are suitable for examining large donor cohorts, some limitations should be noted. First, MDMi do not fully recapitulate the ontogeny of resident microglia, which derive from primitive macrophages in the embryonic yolk sac [64]. However, MDMi can serve as surrogates for infiltrating monocyte-derived macrophages that replace exhausted resident microglia in AD and perform indispensable roles in resolving disease-associated damage in the brain parenchyma [65, 66]. Second, the levels of systemic inflammation, which correlate with AD disease progression [67], influence the state of circulating monocytes. Consequently, the characteristics of MDMi reflect the systemic state of a patient at the time of blood withdrawal and could change as the disease advances. Third, challenges related to the quality and availability of blood samples from AD patients may restrict the generation of MDMi cultures for downstream assays. Nonetheless, it is noteworthy that a recent study successfully performed a high-content drug screen with over 1000 compounds using the MDMi model [51].

In future, the addition of patient hiPSC-derived neural progenitor cells into the 3D MDMi co-cultures would make this platform more personalized, particularly if hiPSCs and MDMi are obtained from the same individual. Such a co-culture model would provide a more accurate representation of the human brain and would allow for modeling disease-specific changes, not possible using ReNcell VM, which derive from a fetal brain and have been immortalized via myc technology [38]. While hiPSC-derived microglial and neural cells better capture ontogeny and more accurately recapitulate the features of these cell types, it is essential to consider the main caveats associated with hiPSCs. These include residual somatic cell epigenetic memory and the potential for de novo epigenetic aberrations [68, 69]. The epigenetic landscape is key to modulate the diversity of microglial functional phenotypes [70], and provides a mechanistic basis for understanding how molecular processes associated with aging may contribute to AD predisposition [71].

## Conclusions

In conclusion, we describe reproducible and easy-to-generate 3D in vitro models of MDMi that can recapitulate potentially important AD-specific differences—not identified in 2D models—associated with diseased microglia. This study opens new doors to generate patient-specific drug testing platforms that support the development of microglia-targeted therapeutic interventions tailored for AD patients and potentially other neurological disorders.

### Supplementary Information

**Additional file 1.** Supplementary figures.

**Additional file 2. Dataset S1.** Top significant differentially expressed genes (DEGs) in individual clusters of combined monocyte, 2D, 3D and 3D co-culture UMAP.

**Additional file 3. Dataset S2.** Average pseudo-bulk expression of common 798 microglial core genes across MDMi models, post-mortem (Olah *et al.*,) and hiPSC-derived (Svoboda *et al.*,) microglia. 

**Additional file 4. Dataset S3.**  Top significant DEGs in clusters of monocytes, 2D, 3D and 3D co-culture individual UMAPs.

**Additional file 5. Dataset S4.** Raw data of morphology parameter analysis and quantification of growth factor and cytokine secretion in AD MDMi models.

**Additional file 6. Table S1.**  qRT-PCT primer sequences.

**Additional file 7. Table S2.**  Correlation analyses between disease-associated phenotypes and *APOE* genotype identified in MDMi from AD patients.

**Additional file 8. Movie S1.**  Rotation of surface reconstruction of Iba1-stained 2D MDMi.

**Additional file 9. Movie S2.**  Rotation of surface reconstruction of Iba1-stained 3D MDMi.

**Additional file 10. Movie S3.**  MDMi migration in 3D co-culture in the presence of FITC-labeled Aβ aggregates.

## Data Availability

All data generated and analyzed to support the findings of this study are included within the article and its additional files. Single-cell RNA sequencing data have been deposited in the NCBI Sequence Read Archive (SRA) with Bioproject accession number PRJNA1067365 ^o^ (https://www.ncbi.nlm.nih.gov/sra/PRJNA1067365). Codes have been deposited in https://github.com/ankit277/scRNA. Additional datasets are available from the corresponding authors upon reasonable request.

## References

[1] Gammon K (2014). Neurodegenerative disease: brain windfall. Nature.

[2] Knopman DS, Jones DT, Greicius MD (2021). Failure to demonstrate efficacy of aducanumab: an analysis of the EMERGE and ENGAGE trials as reported by Biogen, December 2019. Alzheimers Dement.

[3] van Dyck CH, Swanson CJ, Aisen P, Bateman RJ, Chen C, Gee M (2022). Lecanemab in early Alzheimer’s disease. N Engl J Med.

[4] Heneka MT, Carson MJ, El Khoury J, Landreth GE, Brosseron F, Feinstein DL (2015). Neuroinflammation in Alzheimer’s disease. Lancet Neurol.

[5] Jack CR, Knopman DS, Jagust WJ, Shaw LM, Aisen PS, Weiner MW (2010). Hypothetical model of dynamic biomarkers of the Alzheimer’s pathological cascade. Lancet Neurol.

[6] De Strooper B, Karran E (2016). The cellular phase of Alzheimer’s disease. Cell.

[7] Sala Frigerio C, Wolfs L, Fattorelli N, Thrupp N, Voytyuk I, Schmidt I (2019). The major risk factors for Alzheimer’s disease: age, sex, and genes modulate the microglia response to Aβ plaques. Cell Rep.

[8] Sierksma A, Lu A, Mancuso R, Fattorelli N, Thrupp N, Salta E (2020). Novel Alzheimer risk genes determine the microglia response to amyloid-β but not to TAU pathology. EMBO Mol Med.

[9] Stansley B, Post J, Hensley K (2012). A comparative review of cell culture systems for the study of microglial biology in Alzheimer’s disease. J Neuroinflammation.

[10] Haenseler W, Rajendran L (2019). Concise review: modeling neurodegenerative diseases with human pluripotent stem cell-derived microglia. Stem Cells.

[11] Holtman IR, Raj DD, Miller JA, Schaafsma W, Yin Z, Brouwer N (2015). Induction of a common microglia gene expression signature by aging and neurodegenerative conditions: a co-expression meta-analysis. Acta Neuropathol Commun.

[12] Galatro TF, Holtman IR, Lerario AM, Vainchtein ID, Brouwer N, Sola PR (2017). Transcriptomic analysis of purified human cortical microglia reveals age-associated changes. Nat Neurosci.

[13] Gosselin D, Skola D, Coufal NG, Holtman IR, Schlachetzki JCM, Sajti E (2017). An environment-dependent transcriptional network specifies human microglia identity. Science.

[14] Smith AM, Dragunow M (2014). The human side of microglia. Trends Neurosci.

[15] Butovsky O, Jedrychowski MP, Moore CS, Cialic R, Lanser AJ, Gabriely G (2014). Identification of a unique TGF-β–dependent molecular and functional signature in microglia. Nat Neurosci.

[16] Das A, Kim SH, Arifuzzaman S, Yoon T, Chai JC, Lee YS (2016). Transcriptome sequencing reveals that LPS-triggered transcriptional responses in established microglia BV2 cell lines are poorly representative of primary microglia. J Neuroinflammation.

[17] Melief J, Sneeboer M, Litjens M, Ormel P, Palmen S, Huitinga I (2016). Characterizing primary human microglia: a comparative study with myeloid subsets and culture models. Glia.

[18] Sabogal-Guáqueta AM, Marmolejo-Garza A, de Pádua VP, Eggen B, Boddeke E, Dolga AM (2020). Microglia alterations in neurodegenerative diseases and their modeling with human induced pluripotent stem cell and other platforms. Prog Neurobiol.

[19] Mertens J, Reid D, Lau S, Kim Y, Gage FH (2018). Aging in a dish: iPSC-derived and directly induced neurons for studying brain aging and age-related neurodegenerative diseases. Annu Rev Genet.

[20] Sellgren CM, Gracias J, Watmuff B, Biag JD, Thanos JM, Whittredge PB (2019). Increased synapse elimination by microglia in schizophrenia patient-derived models of synaptic pruning. Nat Neurosci.

[21] Ormel PR, Böttcher C, Gigase FAJ, Missall RD, van Zuiden W, Fernández Zapata MC (2020). A characterization of the molecular phenotype and inflammatory response of schizophrenia patient-derived microglia-like cells. Brain Behav Immun.

[22] Ohgidani M, Kato TA, Setoyama D, Sagata N, Hashimoto R, Shigenobu K (2014). Direct induction of ramified microglia-like cells from human monocytes: dynamic microglial dysfunction in Nasu-Hakola disease. Sci Rep.

[23] Quek H, Cuní-López C, Stewart R, Colletti T, Notaro A, Nguyen TH (2022). ALS monocyte-derived microglia-like cells reveal cytoplasmic TDP-43 accumulation, DNA damage, and cell-specific impairment of phagocytosis associated with disease progression. J Neuroinflammation.

[24] Ryan KJ, White CC, Patel K, Xu J, Olah M, Replogle JM (2017). A human microglia-like cellular model for assessing the effects of neurodegenerative disease gene variants. Sci Transl Med.

[25] Penney J, Ralvenius WT, Tsai L-H (2020). Modeling Alzheimer’s disease with iPSC-derived brain cells. Mol Psychiatry.

[26] Watson PMD, Kavanagh E, Allenby G, Vassey M (2017). Bioengineered 3D glial cell culture systems and applications for neurodegeneration and neuroinflammation. SLAS Discovery.

[27] Abud EM, Ramirez RN, Martinez ES, Healy LM, Nguyen CHH, Newman SA (2017). iPSC-derived human microglia-like cells to study neurological diseases. Neuron.

[28] Choi SH, Kim YH, Hebisch M, Sliwinski C, Lee S, D’Avanzo C (2014). A three-dimensional human neural cell culture model of Alzheimer’s disease. Nature.

[29] D’avanzo C, Aronson J, Kim YH, Choi SH, Tanzi RE, Kim DY (2015). Alzheimer’s in 3D culture: challenges and perspectives. BioEssays.

[30] Bennett ML, Bennett FC, Liddelow SA, Ajami B, Zamanian JL, Fernhoff NB (2016). New tools for studying microglia in the mouse and human CNS. Proc Natl Acad Sci U S A.

[31] Lupton MK, Robinson GA, Adam RJ, Rose S, Byrne GJ, Salvado O (2021). A prospective cohort study of prodromal Alzheimer’s disease: prospective imaging study of ageing: genes, brain and behaviour (PISA). NeuroImage Clin.

[32] Quek H, Cuní-López C, Stewart R, Lim YC, Roberts TL, White AR (2022). A robust approach to differentiate human monocyte-derived microglia from peripheral blood mononuclear cells. STAR Protoc.

[33] Olah M, Menon V, Habib N, Taga MF, Ma Y, Yung CJ (2020). Single cell RNA sequencing of human microglia uncovers a subset associated with Alzheimer’s disease. Nat Commun.

[34] Svoboda DS, Barrasa MI, Shu J, Rietjens R, Zhang S, Mitalipova M (2019). Human iPSC-derived microglia assume a primary microglia-like state after transplantation into the neonatal mouse brain. Proc Natl Acad Sci U S A.

[35] Kim YH, Choi SH, D’Avanzo C, Hebisch M, Sliwinski C, Bylykbashi E (2015). A 3D human neural cell culture system for modeling Alzheimer’s disease. Nat Protoc.

[36] Oikari LE, Pandit R, Stewart R, Cuní-López C, Quek H, Sutharsan R (2020). Altered brain endothelial cell phenotype from a familial Alzheimer mutation and its potential implications for amyloid clearance and drug delivery. Stem Cell Reports.

[37] Young K, Morrison H (2018). Quantifying microglia morphology from photomicrographs of immunohistochemistry prepared tissue using ImageJ. J Vis Exp.

[38] Donato R, Miljan EA, Hines SJ, Aouabdi S, Pollock K, Patel S (2007). Differential development of neuronal physiological responsiveness in two human neural stem cell lines. BMC Neurosci.

[39] Patir A, Shih B, McColl BW, Freeman TC (2019). A core transcriptional signature of human microglia: Derivation and utility in describing region-dependent alterations associated with Alzheimer’s disease. Glia.

[40] Buttgereit A, Lelios I, Yu X, Vrohlings M, Krakoski NR, Gautier EL (2016). Sall1 is a transcriptional regulator defining microglia identity and function. Nat Immunol.

[41] Rangaraju S, Dammer EB, Raza SA, Rathakrishnan P, Xiao H, Gao T (2018). Identification and therapeutic modulation of a pro-inflammatory subset of disease-associated-microglia in Alzheimer’s disease. Mol Neurodegener.

[42] Han KH, Arlian BM, Macauley MS, Paulson JC, Lerner RA (2018). Migration-based selections of antibodies that convert bone marrow into trafficking microglia-like cells that reduce brain amyloid β. Proc Natl Acad Sci U S A.

[43] Masuda T, Sankowski R, Staszewski O, Böttcher C, Amann L, Sagar (2019). Spatial and temporal heterogeneity of mouse and human microglia at single-cell resolution. Nature.

[44] Rajendran L, Paolicelli RC (2018). Microglia-mediated synapse loss in Alzheimer’s disease. J Neurosci.

[45] Ryu K-Y, Lee H-J, Woo H, Kang R-J, Han K-M, Park H (2019). Dasatinib regulates LPS-induced microglial and astrocytic neuroinflammatory responses by inhibiting AKT/STAT3 signaling. J Neuroinflammation.

[46] Zheng LT, Hwang J, Ock J, Lee MG, Lee W-H, Suk K (2008). The antipsychotic spiperone attenuates inflammatory response in cultured microglia via the reduction of proinflammatory cytokine expression and nitric oxide production. J Neurochem.

[47] Dhawan G, Combs CK (2012). Inhibition of Src kinase activity attenuates amyloid associated microgliosis in a murine model of Alzheimer’s disease. J Neuroinflammation.

[48] Zúñiga Santamaría T, Yescas Gómez P, Fricke Galindo I, González González M, Ortega Vázquez A, López LM (2022). Pharmacogenetic studies in Alzheimer disease. Neurologia (Engl Ed).

[49] King A (2018). The search for better animal models of Alzheimer’s disease. Nature.

[50] Ohgidani M, Kato TA, Kanba S (2015). Introducing directly induced microglia-like (iMG) cells from fresh human monocytes: a novel translational research tool for psychiatric disorders. Front Cell Neurosci.

[51] Connor SM, Rashid M, Ryan KJ, Patel K, Boyd JD, Smith J (2022). GW5074 increases microglial phagocytic activities: potential therapeutic direction for Alzheimer’s disease. Front Cell Neurosci.

[52] Park J, Wetzel I, Marriott I, Dréau D, D’Avanzo C, Kim DY (2018). A 3D human triculture system modeling neurodegeneration and neuroinflammation in Alzheimer’s disease. Nat Neurosci.

[53] Haw RTY, Tong CK, Yew A, Lee HC, Phillips JB, Vidyadaran S (2014). A three-dimensional collagen construct to model lipopolysaccharide-induced activation of BV2 microglia. J Neuroinflammation.

[54] Cserép C, Pósfai B, Lénárt N, Fekete R, László ZI, Lele Z (2020). Microglia monitor and protect neuronal function through specialized somatic purinergic junctions. Science.

[55] Assaraf MI, Diaz Z, Liberman A, Miller WH, Arvanitakis Z, Li Y (2007). Brain erythropoietin receptor expression in Alzheimer disease and mild cognitive impairment. J Neuropathol Exp Neurol.

[56] Masliah E, Mallory M, Alford M, Deteresa R, Saitoh T (1995). PDGF is associated with neuronal and glial alterations of Alzheimer’s disease. Neurobiol Aging.

[57] Raman MR, Himali JJ, Conner SC, DeCarli C, Vasan RS, Beiser AS (2018). Circulating vascular growth factors and magnetic resonance imaging markers of small vessel disease and atrophy in middle-aged adults. Stroke.

[58] Tarkowski E, Wallin A, Regland B, Blennow K, Tarkowski A (2001). Local and systemic GM-CSF increase in Alzheimer’s disease and vascular dementia. Acta Neurol Scand.

[59] Belkhelfa M, Rafa H, Medjeber O, Arroul-Lammali A, Behairi N, Abada-Bendib M (2014). IFN-γ and TNF-α are involved during Alzheimer disease progression and correlate with nitric oxide production: a study in Algerian patients. J Interferon Cytokine Res.

[60] Lai AY, McLaurin J (2012). Clearance of amyloid-β peptides by microglia and macrophages: the issue of what, when and where. Future Neurol.

[61] Bolmont T, Haiss F, Eicke D, Radde R, Mathis CA, Klunk WE (2008). Dynamics of the microglial/amyloid interaction indicate a role in plaque maintenance. J Neurosci.

[62] Zietarska M, Maugard CM, Filali-Mouhim A, Alam-Fahmy M, Tonin PN, Provencher DM (2007). Molecular description of a 3D in vitro model for the study of epithelial ovarian cancer (EOC). Mol Carcinog.

[63] Souza AG, Silva IBB, Campos-Fernandez E, Barcelos LS, Souza JB, Marangoni K (2018). Comparative assay of 2D and 3D cell culture models: proliferation, gene expression and anticancer drug response. Curr Pharm Des.

[64] Ginhoux F, Greter M, Leboeuf M, Nandi S, See P, Gokhan S (2010). Fate mapping analysis reveals that adult microglia derive from primitive macrophages. Science.

[65] Butovsky O, Kunis G, Koronyo-Hamaoui M, Schwartz M (2007). Selective ablation of bone marrow-derived dendritic cells increases amyloid plaques in a mouse Alzheimer’s disease model. Eur J Neurosci.

[66] Shechter R, London A, Varol C, Raposo C, Cusimano M, Yovel G (2009). Infiltrating blood-derived macrophages are vital cells playing an anti-inflammatory role in recovery from spinal cord injury in mice. PLoS Med.

[67] Holmes C, Cunningham C, Zotova E, Woolford J, Dean C, Kerr S (2009). Systemic inflammation and disease progression in Alzheimer disease. Neurology.

[68] Ruiz S, Diep D, Gore A, Panopoulos AD, Montserrat N, Plongthongkum N (2012). Identification of a specific reprogramming-associated epigenetic signature in human induced pluripotent stem cells. Proc Natl Acad Sci U S A.

[69] Kim K, Doi A, Wen B, Ng K, Zhao R, Cahan P (2010). Epigenetic memory in induced pluripotent stem cells. Nature.

[70] Zhang X, Kracht L, Lerario AM, Dubbelaar ML, Brouwer N, Wesseling EM (2022). Epigenetic regulation of innate immune memory in microglia. J Neuroinflammation.

[71] Nativio R, Donahue G, Berson A, Lan Y, Amlie-Wolf A, Tuzer F (2018). Dysregulation of the epigenetic landscape of normal aging in Alzheimer’s disease. Nat Neurosci.

