# Correction: Advanced patient-specific microglia cell models for pre-clinical studies in Alzheimer’s disease

**DOI:** 10.1186/s12974-024-03074-y

**Published:** 2024-04-10

**Authors:** Carla Cuní‑López, Romal Stewart, Lotta E. Oikari, Tam Hong Nguyen, Tara L. Roberts, Yifan Sun, Christine C. Guo, Michelle K. Lupton, Anthony R. White, Hazel Quek

**Affiliations:** 1 Mental Health and Neuroscience Department, QIMR Berghofer Medical Research Institute, Herston, QLD 4006 Australia; 2 Faculty of Medicine, The University of Queensland, Herston, QLD 4006 Australia; 3 School of Biomedical Sciences, The University of Queensland, Lucia, QLD 4072 Australia; 4 School of Biomedical Sciences, Queensland University of Technology, Brisbane City, QLD 4000 Australia; 5 UQ Centre for Clinical Research, The University of Queensland, Brisbane City, QLD 4029 Australia; 6 Scientific Services, QIMR Berghofer Medical Research Institute, Herston, QLD 4006 Australia; 7 Ingham Institute for Applied Medical Research and School of Medicine, Western Sydney University, Liverpool, NSW 2170 Australia; 8 Present Address: ActiGraph LLC, Pensacola, FL 32502 USA

**Correction to: Journal of Neuroinflammation (2024) 21:50**

10.1186/s12974-024-03037-3

In this article [1], there is an error in the “Availability of data and materials” section. The SRA link in the current version of the manuscript is no longer functional and should be updated with the GEO ID provided below.

## Data Availability

All data generated and analyzed to support the findings of this study are included within the article and its additional files. Single-cell RNA sequencing data have been deposited in the Gene Expression Omnibus (GEO) under accession code GSE255718. The analysis codes have been deposited in https://github.com/ankit277/scRNA. Additional datasets are available from the corresponding authors upon reasonable request.
