# Supplementary material for: Advanced patient-specific microglia cell models for pre-clinical studies in Alzheimer’s disease
Source: J Neuroinflammation. 2024 Feb 15;21:50. doi: 10.1186/s12974-024-03037-3 (PMC10870454; doi:10.1186/s12974-024-03037-3)
Supplement: Supplementary file 1 — Additional file 1. Supplementary figures. [file 12974_2024_3037_MOESM1_ESM.docx]

**Additional file 1**

**
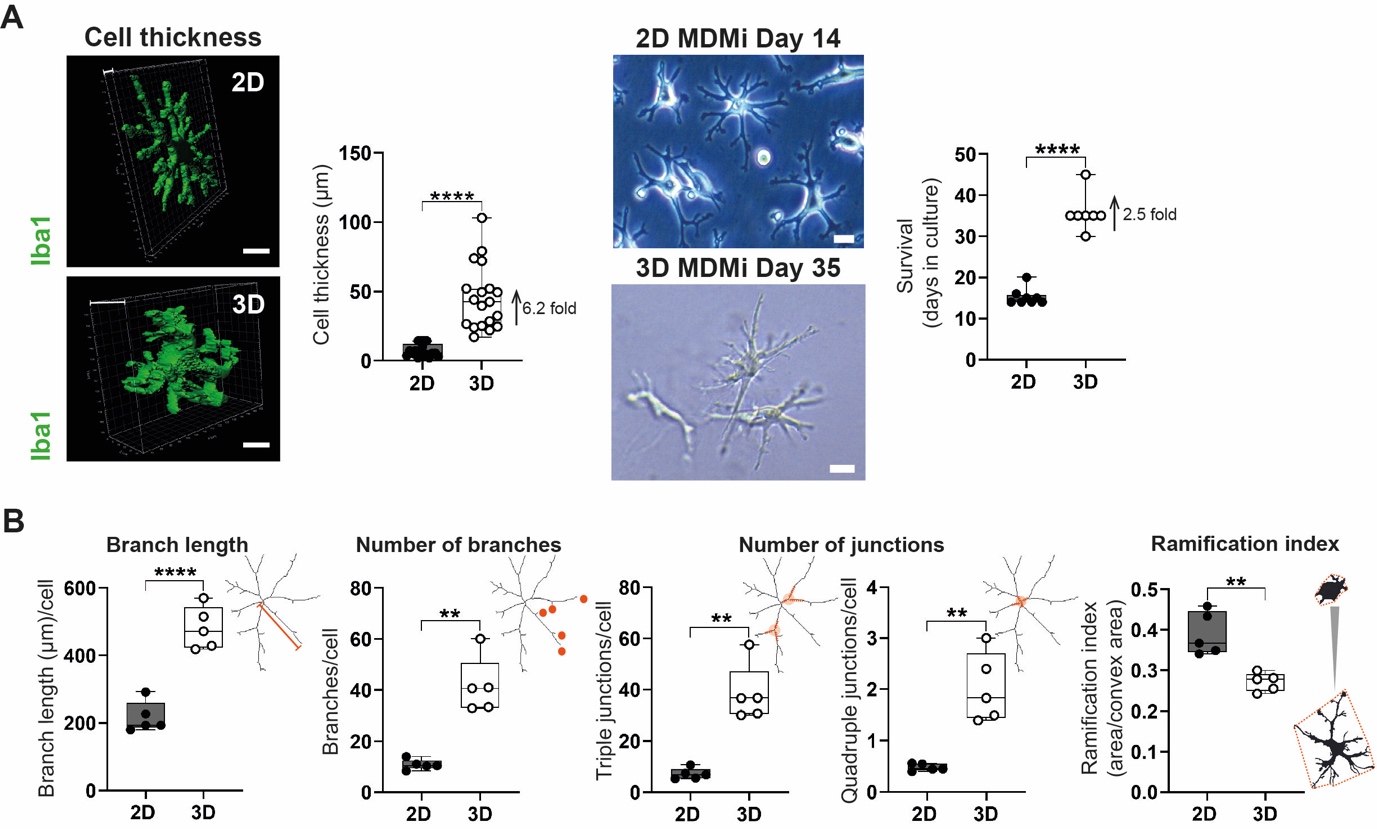
**

**Fig. S1.** **3D MDMi show increased cell thickness, survival in culture and ramified morphology compared to 2D. (A)** 3D surface rendered images of Iba1-stained MDMi and thickness of mono-cultures in 2D and 3D (see Movies S1, 2). Representative bright field images of 2D and 3D MDMi after 14 (top) or 35 (bottom) days of differentiation, respectively, and quantification of cell survival as number of days the cells were alive in in culture (*n* = 8 independent experiments for 2D, *n* = 7 independent experiments for 3D). Scale bars, 25 μm. **(B)** Quantification of morphological parameters in 2D (*n* = 5) and 3D MDMi (*n* = 5), including branch length, number of branches, number of junctions ­—including triple junctions (left) and quadruple junctions (right)— and ramification index (area/convex area). Representative skeleton and binary images are included on the right of each graph to illustrate the morphological measurements. Data are presented as mean ± SD. Data points and *n* represent biological replicates (donors). Unpaired Student’s *t* test with or without Welch’s correction, two-tailed; ***P* < 0.01, *****P* < 0.0001.


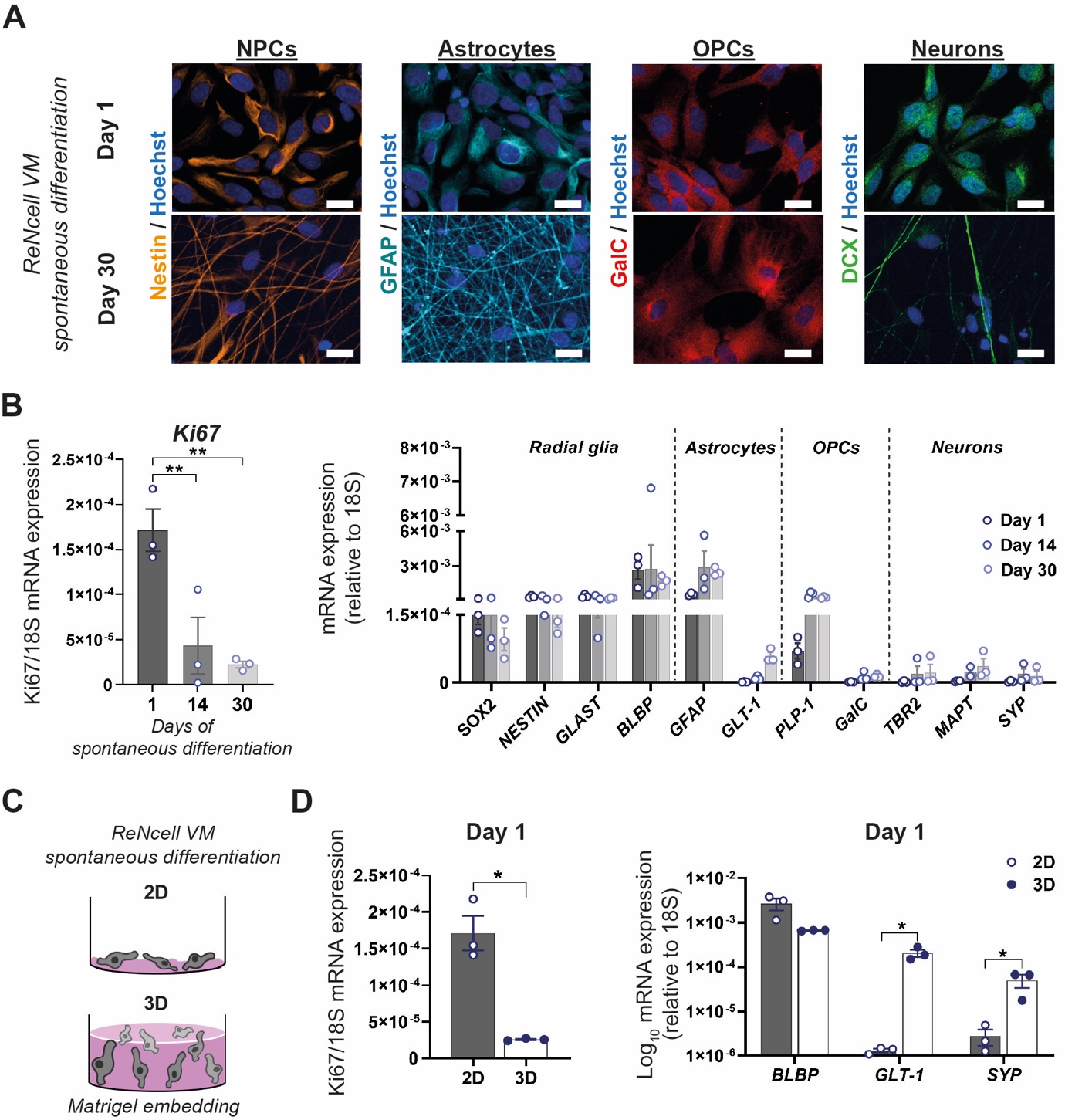


**Fig. S2.** **Characterization of differentiated ReNcell VM in 2D and 3D mono-cultures. (A)** Immunostaining of 2D ReNcell VM cultures differentiated for 1 and 30 days shows expression of characteristic markers of neural progenitor cells (NPCs; Nestin), astrocytes (GFAP), oligodendrocyte progenitor cells (OPCs; GalC) and neurons (DCX). Scale bars, 100 μm. **(B)** mRNA expression of the proliferation marker *Ki67*, and a panel of radial glia (NPCs), astrocytes, OPCs and neuron markers in 2D ReNcell VM cultures spontaneously differentiated for 1, 14 or 30 days (*n* = 3 independent experiments). **(C)** Schematic of ReNcell VM cultures undergoing spontaneous differentiation for 1 day in 2D, or in 3D upon embedment in Matrigel. **(D)** mRNA expression of *Ki67* alongside the radial glia marker *BLBP*, the mature astrocyte marker *GLT-1* and the mature neuron marker *SYP* in 2D (*n* = 3 independent experiments) and 3D (*n* = 3 independent experiments) ReNcell VM cultures spontaneously differentiated for 1 day. Data are presented as mean ± SEM. One-way ANOVA with Tukey’s multiple comparison test in **B**; unpaired Student’s *t* test with or without Welch’s correction, two-tailed in **D**; **P* < 0.05, ***P* < 0.01.


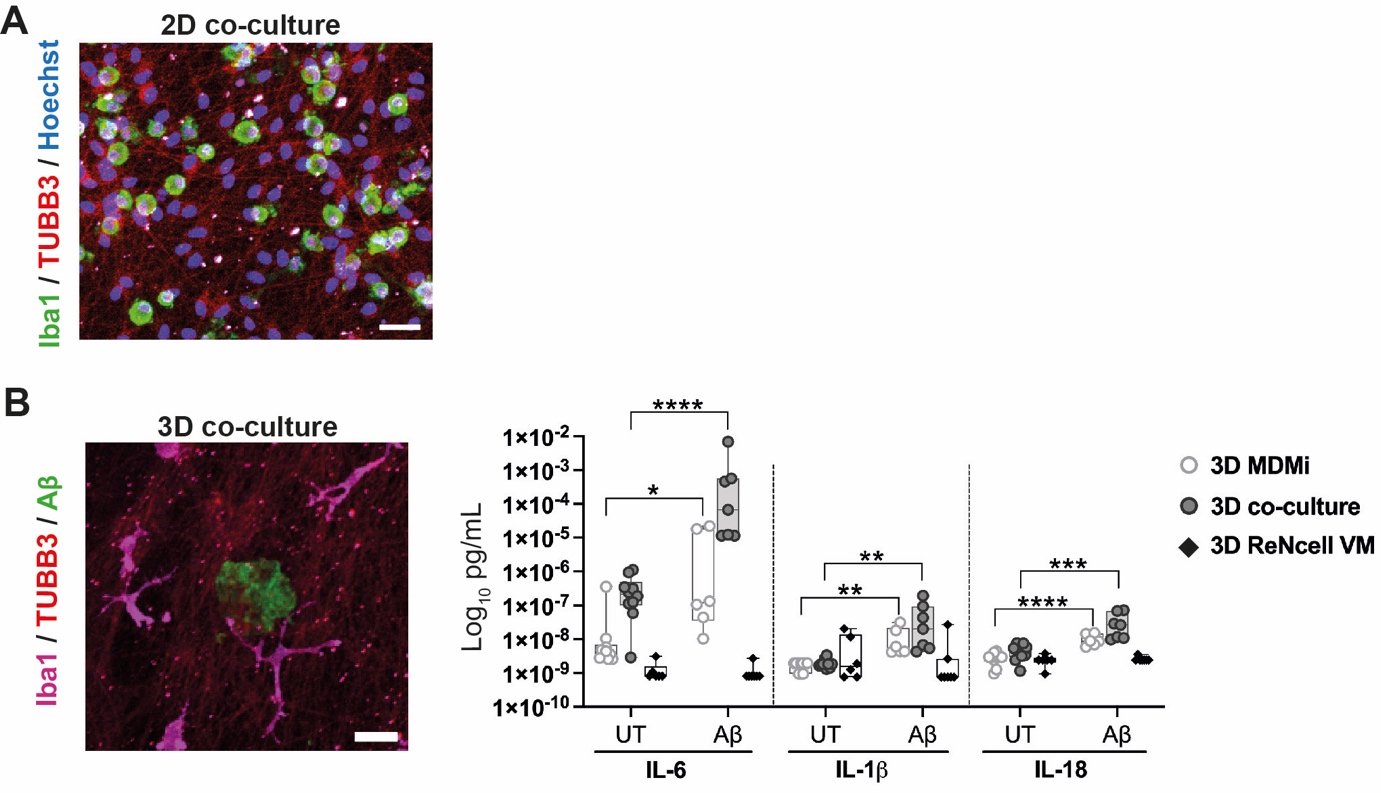


**Fig. S3.** **MDMi only differentiate in 3D co-cultures with ReNcell VM and elicit and inflammatory response to Aβ. (A)** MDMi co-culture with ReNcell VM in 2D is insufficient for monocyte differentiation into MDMi, as monocytes retain a round morphology after 40 days in 2D co-culture. MDMi were stained for Iba1 and ReNcell VM were stained for TUBB3. Scale bar, 100 μm. **(B)** Immunofluorescence image of 3D co-cultures containing FITC-Aβ aggregates. Scale bar, 100 μm. Concentration of secreted pro-inflammatory cytokines IL-6, IL-1β and IL-18 by 3D MDMi (*n* = 9-10 untreated (UT) cultures; *n* = 6 Aβ-treated cultures), ReNcell VM (*n* = 6 UT cultures; *n* = 7 Aβ-treated cultures) mono-cultures and 3D co-cultures (*n* = 10 UT cultures; *n* = 7 Aβ-treated cultures) upon exposure to FITC-Aβ aggregates. Data are presented as mean ± SD. Data points and *n* represent biological replicates (donors) for 3D MDMi and 3D co-culture, and independent experiments for 3D ReNcell VM. Unpaired Student’s *t* test with or without Welch’s correction, two-tailed; **P* < 0.05, ***P* < 0.01, ****P* < 0.001, *****P* < 0.0001.


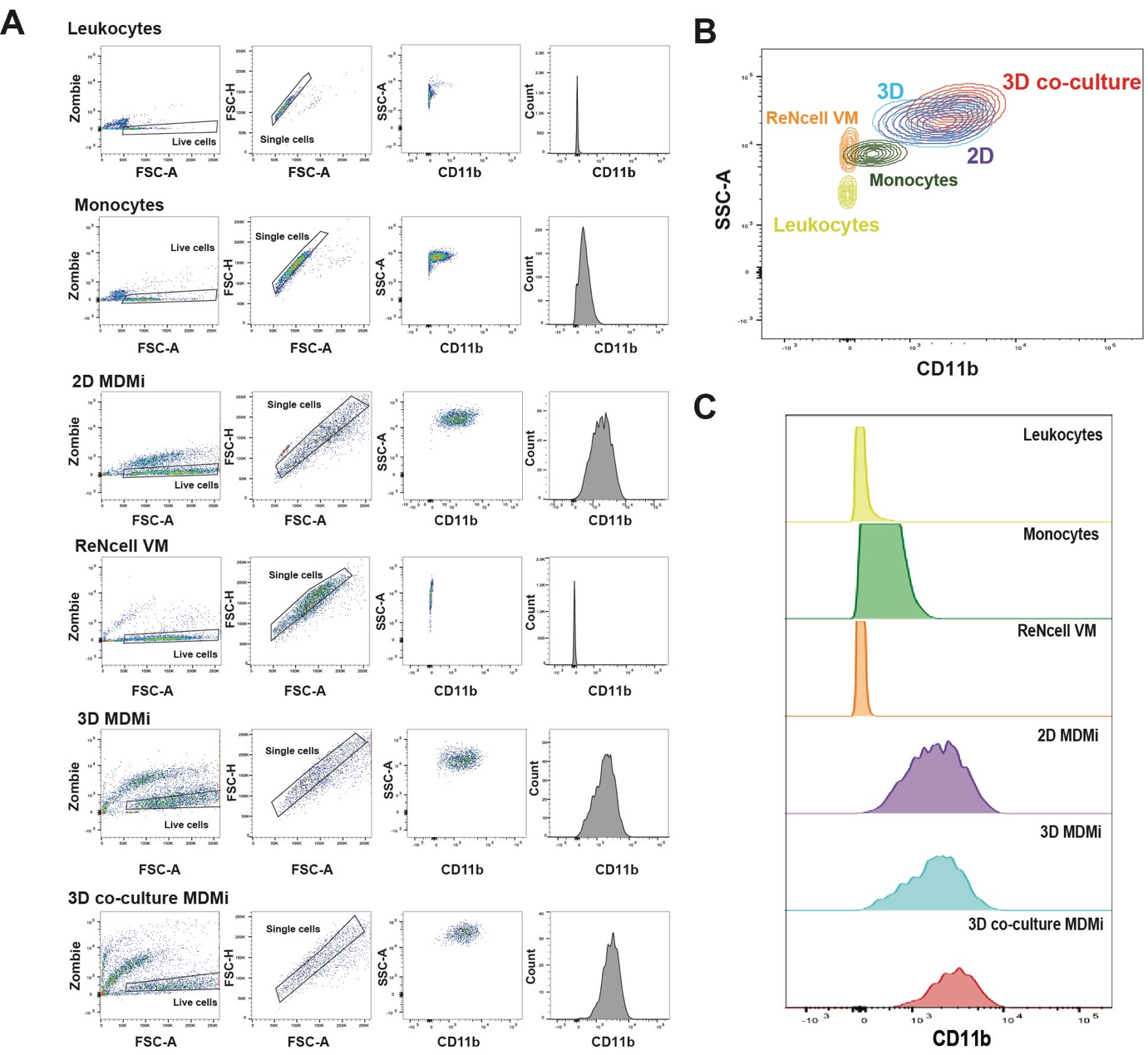


**Fig. S4. Gating strategy for FACs sorting of monocytes and MDMi in 2D, 3D and 3D co-culture. (A)** FACs gating strategy for sorting MDMi. Density dot plots of each culture platform (2D, 3D and 3D co-cultures) and relevant controls (leukocytes, monocytes and ReNcell VM). Live cells were identified by ZombieAqua^negative^ and forward scatter (FSC), and cell doublets were removed by FSC-A and FSC-H gating. From these cells, we identified MDMi subsets based on sideward scatter (SSC) and CD11b staining in **(B)** for discrimination within each platform. Contour plot showing the discrimination strategy of different cell types by their CD11b expression and SSC-A gating. **(C)** Low CD11b expression in leukocytes, monocytes and ReNcells VM was observed while CD11b expression was high in all MDMi cultures (2D, 3D and 3D co-culture).

**
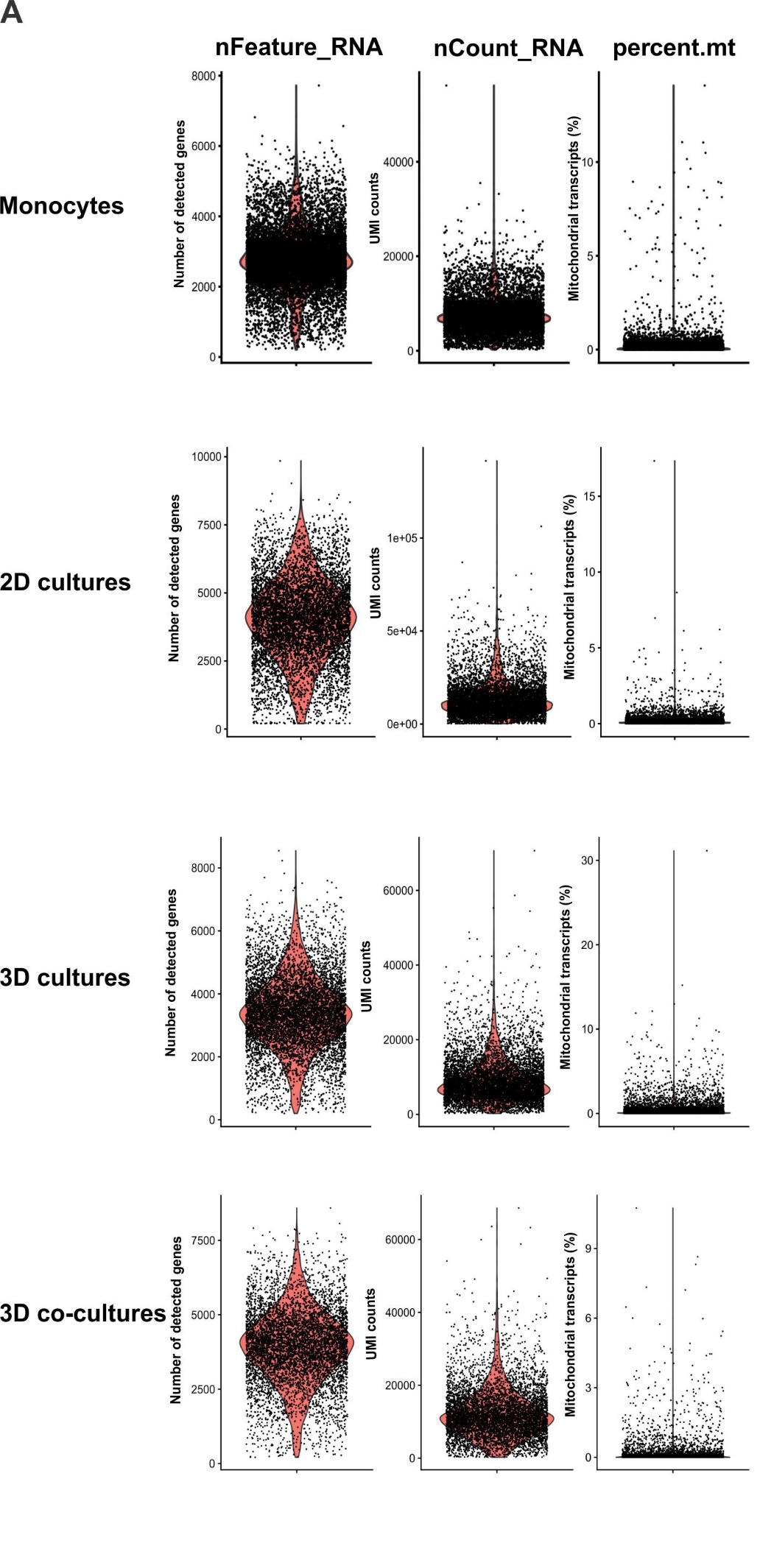
**

**Fig. S5. scRNAseq quality of cells across MDMi models. (A)** Violin plots displaying the number of detected genes, UMI counts and percentage mitochondrial transcripts. UMI: unique molecular identifier.

**
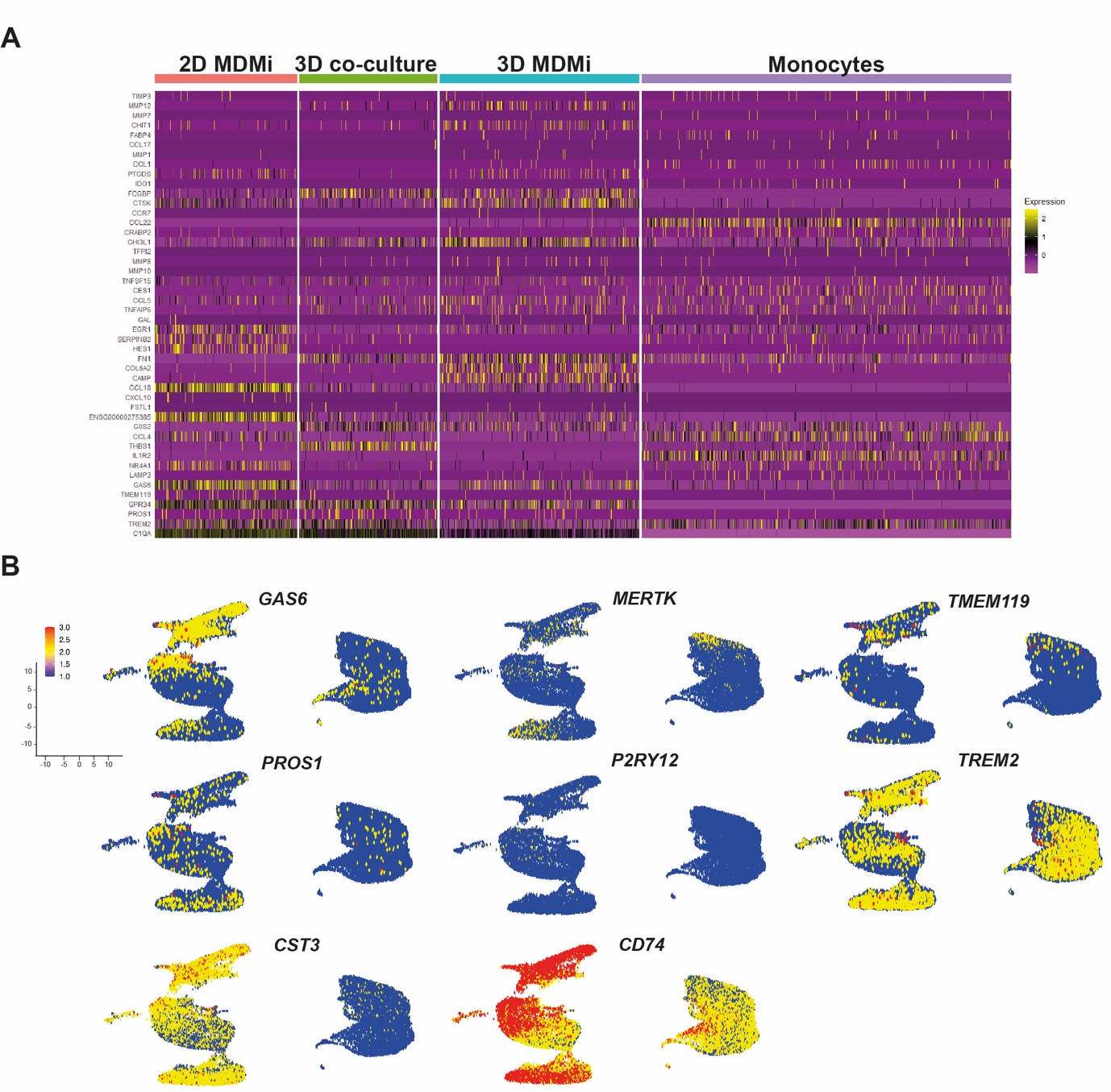
**

**Fig. S6. scRNAseq shows top variable genes across monocytes and all MDMi models. (A)** Top 40 variable genes from Fig. 1D examined across monocytes and MDMi models, and their expression levels are projected onto a heat map. Y-axis represents the top variable genes and X-axis represents individual cells from each model. Highly expressed genes are visualized in yellow and lowly expressed genes are visualized in purple. **(B)** Combined UMAP plots of monocytes and MDMi (2D, 3D and 3D co-culture) show the expression of selected myeloid and/or microglia genes. Each dot represents a cell and the normalized gene expression levels of the selected genes for each cell. The color gradient bar represents log-transformed expression values, with red and blue indicating maximum and minimum expression, respectively.


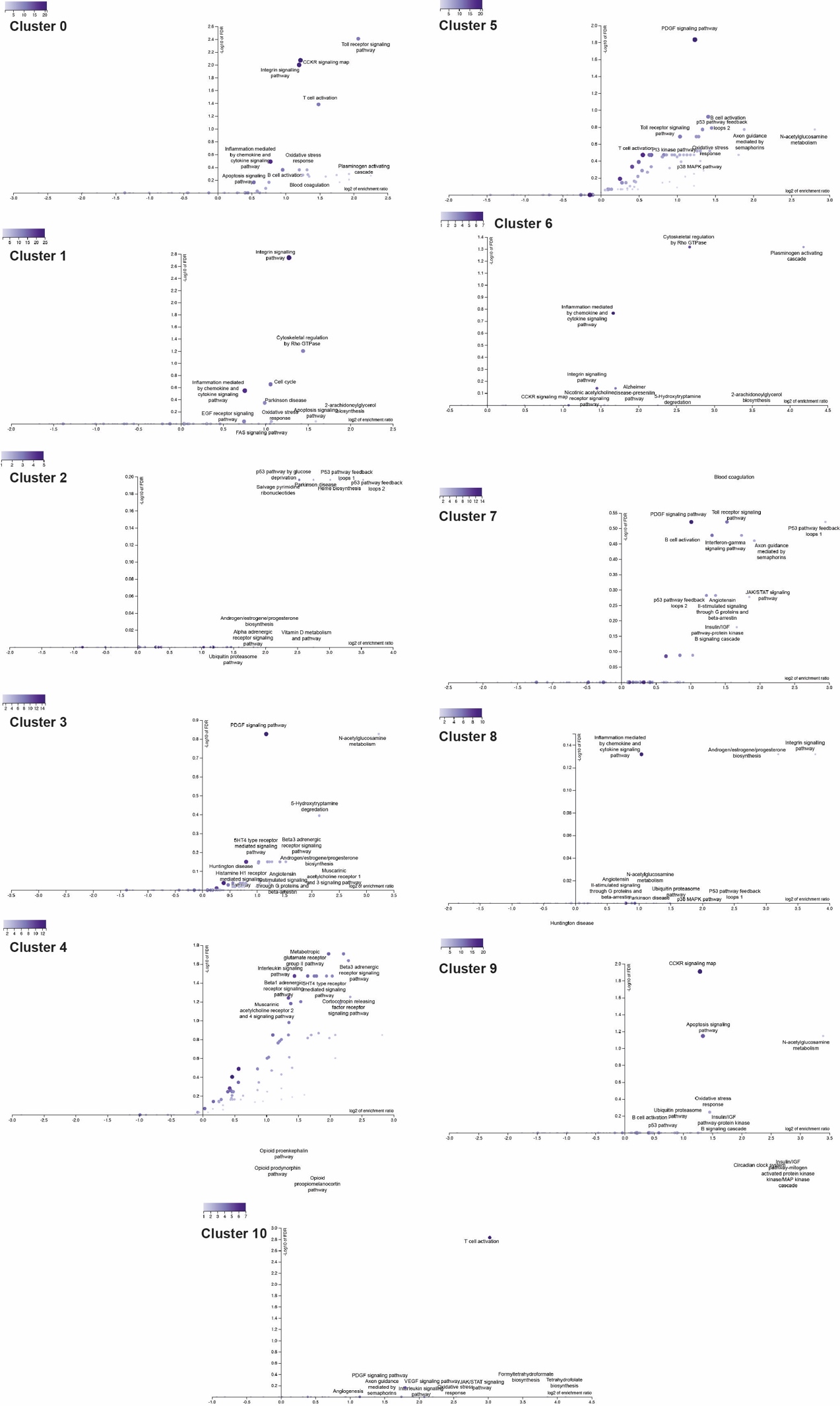


**Fig. S7. Pathway enrichment analysis for combined UMAP of all models.** Pathway enrichment analysis of individual clusters (0-10) identified in Fig. 1H, I. The statistical test used was the hypergeometric test, and the correction methods for multiple testing were Benjamini-Hochberg and FDR. Significance level: *P*-value threshold of 0.05.


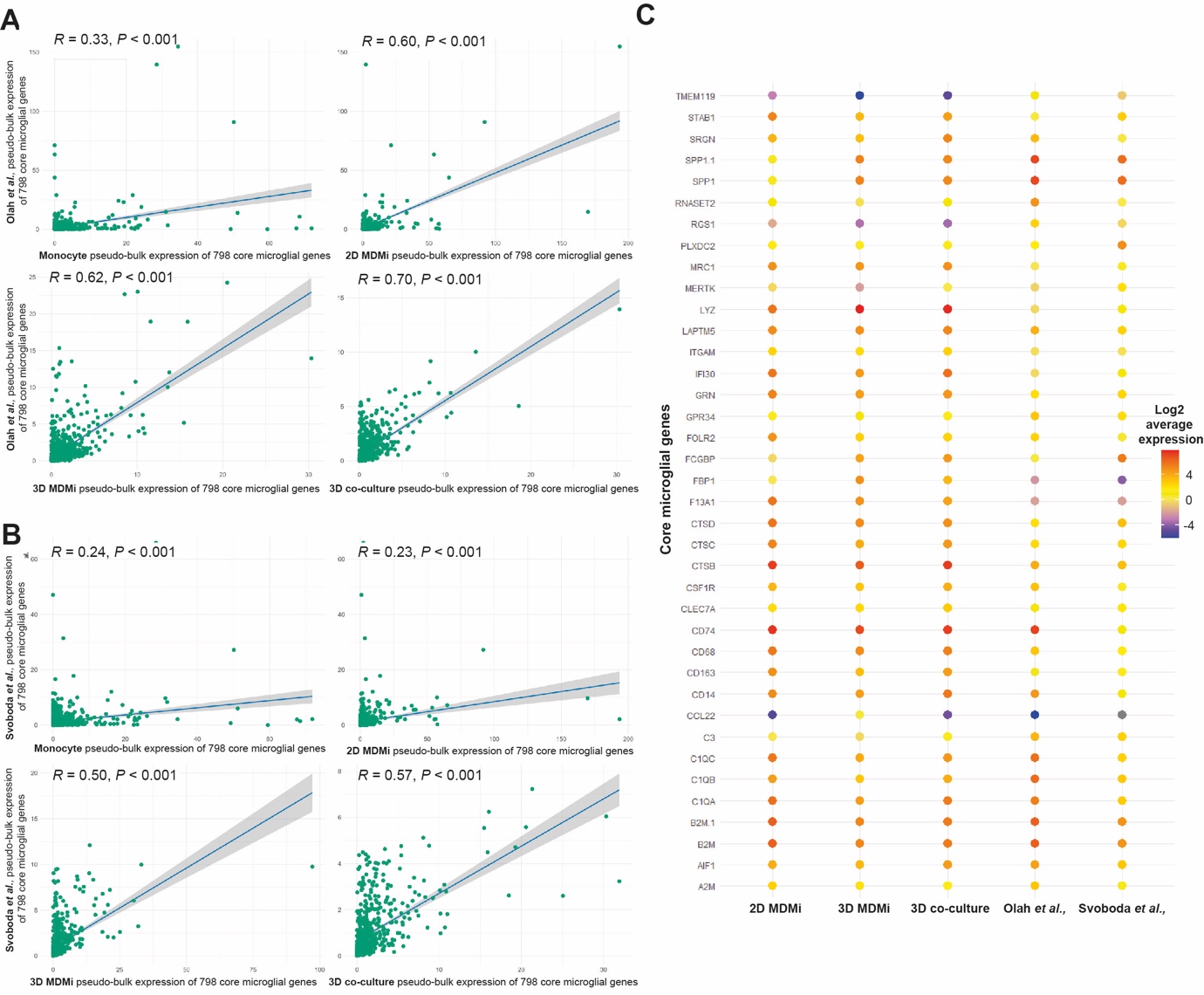


**Fig. S8. Pseudo-bulk expression profiles of core microglial genes in MDMi models benchmarked against published datasets of post-mortem and hiPSC-derived microglia.** Correlation tests of pseudo-bulk expression of 789 core microglia genes between monocytes and MDMi compared with **(A)** post-mortem microglia (Olah *et al.,*) and **(B)** hiPSC-derived microglia (Svoboda *et al.,*). Spearman correlation coefficient (R) was used to quantify the degree of correlation. **(C)** Average expression levels of the most variable 38 core microglial genes across MDMi models. With this selection, differences between models are more evident, thereby facilitating the identification of the MDMi culture system that best captures the transcriptomic signature of benchmark microglia models.


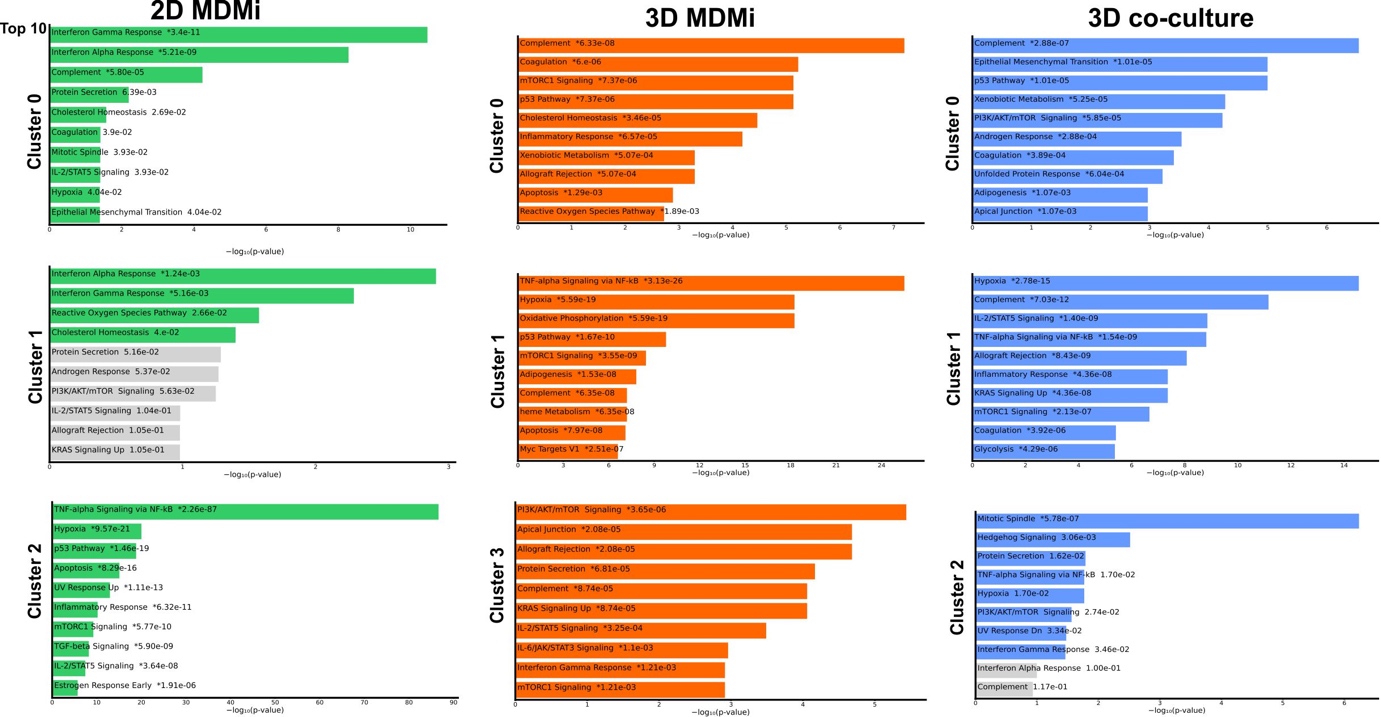


**Fig. S9. Bar chart of top enriched terms in MDMi (2D, 3D, and 3D co-culture) individual clusters.** Bar chart of top enriched terms from the MSigDB gene set library. The top 10 enriched terms for the input gene set are displayed based on the -log10(p-value), with the actual *P*-values shown next to each term. The term at the top has the most significant overlap with the input query gene set. A (*) next to a *P*-value indicates that the pathway also has a significant adjusted *P*-value (<0.05).

**
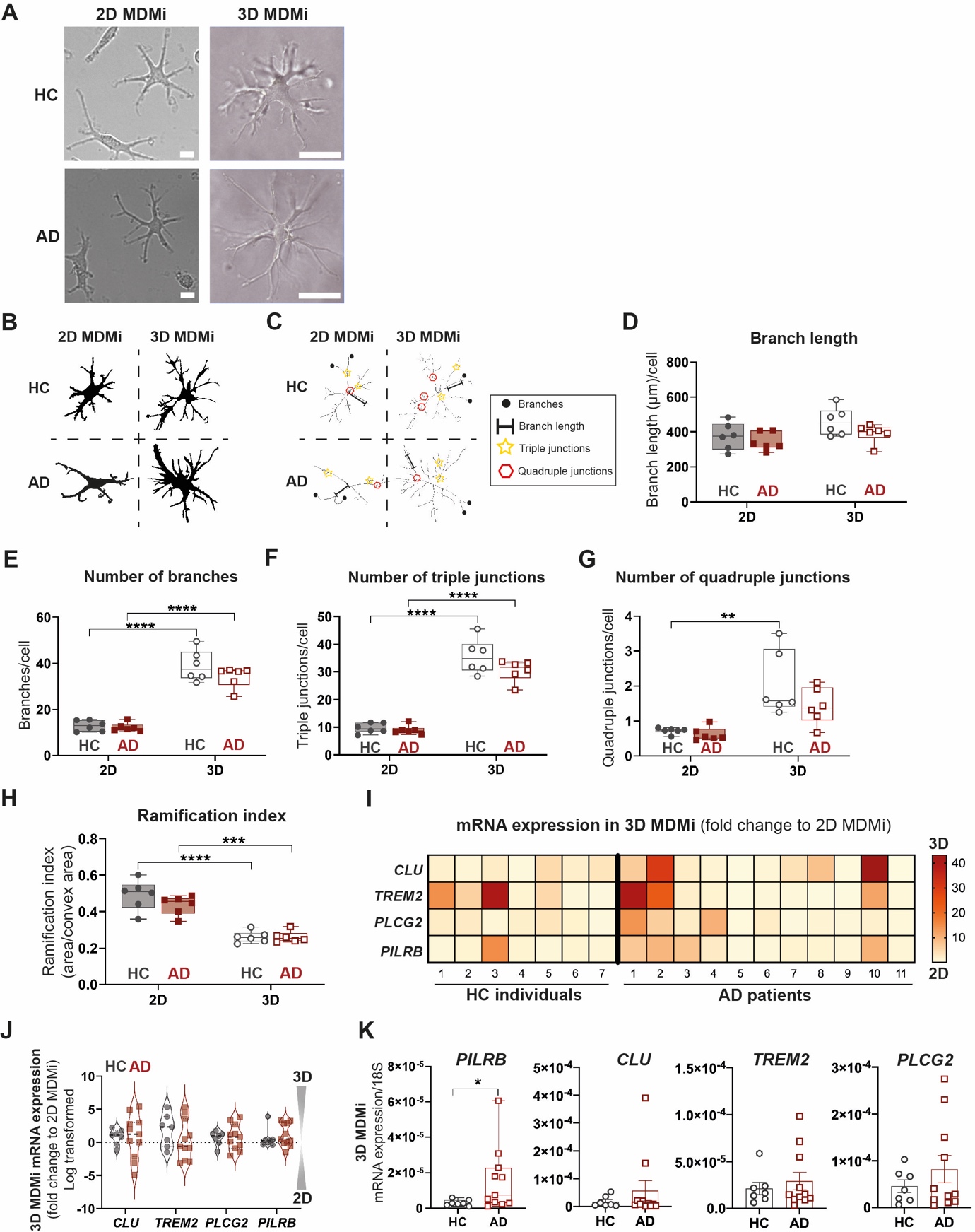
**

**Fig. S10.** **AD-associated phenotypes in 2D and 3D MDMi mono-cultures related to morphology and expression of AD risk genes.** **(A)** Representative bright field images of HC and AD MDMi in 2D and 3D mono-cultures. Scale bars, 100 μm. **(B)** Binary and **(C)** skeleton images of HC and AD MDMi in 2D and 3D mono-cultures showing the morphological parameters (branch number, branch length, triple and quadruple junctions) analyzed. Quantification of morphological parameters from both HC (*n* = 6) and AD (*n* = 6) cohorts, including **(D)** branch length, **(E)** number of branches, **(F)** number of triple junctions, **(G)** number of quadruple junctions and **(H)** ramification index. **(I)** Heat map representing HC (*n* = 7) and AD (*n* = 11) individual-specific fold changes in gene expression levels of the AD risk genes *CLU*, *TREM2*, *PLCG2* and *PILRB* in 3D MDMi compared to 2D. Red-yellow color spectrum shows relative fold change of 3D MDMi as compared to 2D MDMi. **(J)** Violin plot representation of fold change (log-transformed) of mRNA expression in 3D to 2D HC (*n* = 7) and AD (*n* = 11) MDMi. **(K)** mRNA expression of the AD risk genes *PILRB, CLU*, *TREM2*, *PLCG2* in 3D HC (*n* = 7) and AD (*n* = 11) MDMi. Data are presented as mean ± SD. Data points and *n* represent biological replicates (donors). Two-way ANOVA with Šídák's multiple comparison test in **E-H**; unpaired Student’s *t* test with or without Welch’s correction, two-tailed in **K**; **P* < 0.05, ***P* < 0.01, ****P* < 0.001, *****P* < 0.0001.


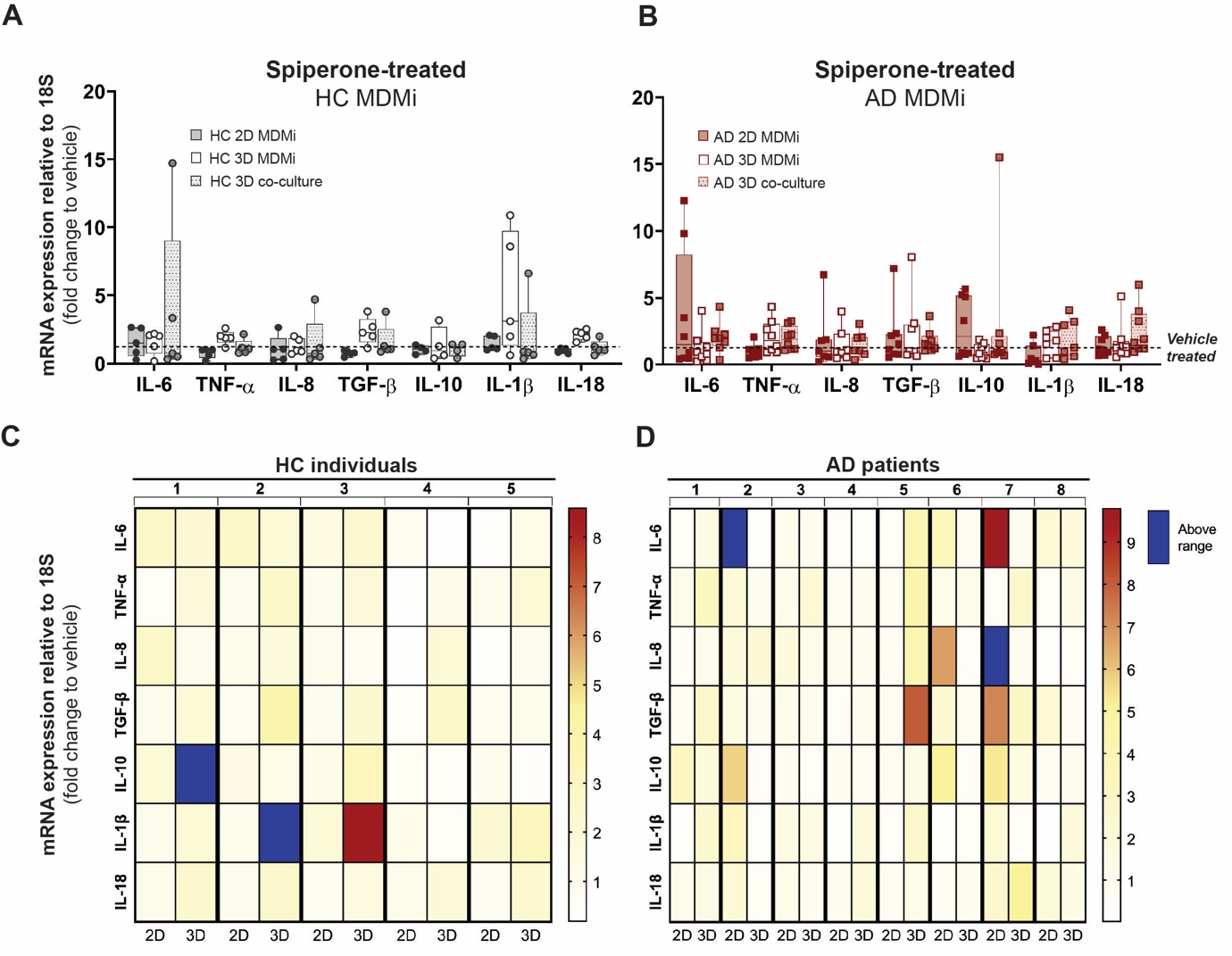


**Fig. S11. Spiperone treatment induces donor- and model-specific cytokine responses. (A, B)** Fold change in cytokine mRNA expression levels following 24 h exposure to 1 µM spiperone compared to vehicle (DMSO)-treated cultures in HC (*n* = 4-5) and AD (*n* = 7-8) MDMi (2D, 3D and 3D co-cultures). Dotted black lines represent baseline responses of vehicle-treated cultures. **(C, D)** Heat maps showing HC (*n* = 5) and AD (*n* = 8) donor-specific changes in mRNA expression from 2D and 3D MDMi mono-cultures. Red-yellow color spectrum represents relative fold change of mRNA expression after spiperone treatment compared to vehicle. Expression changes falling outside the displayed range are indicated in dark blue. Data are presented as mean ± SEM. Data points and *n* represent biological replicates (donors). One-way ANOVA with Dunnett’s multiple comparison test.
