# Supplementary material for: Advanced patient-specific microglia cell models for pre-clinical studies in Alzheimer’s disease
Source: J Neuroinflammation. 2024 Feb 15;21:50. doi: 10.1186/s12974-024-03037-3 (PMC10870454; doi:10.1186/s12974-024-03037-3)
Supplement: Supplementary file 6 — Additional file 6. Table S1. qRT-PCT primer sequences. [file 12974_2024_3037_MOESM6_ESM.docx]

**Table S1. qRT-PCR primer sequences.**

| **Primer** | **Forward sequence** | **Reverse sequence** | **Size (bp)** |
| --- | --- | --- | --- |
| *18S* | TTCGAGGCCCTGTAATTGGA | GCAGCAACTTTAATATACGCTATTGG | 123 |
| *TREM2* | TCTTTGTCACAGAGCTGTCC | TCATAGGGGCAAGACACCTG | 88 |
| *KI67* | gaggtgtgcagaaaatccaaa | ctgtccctatgacttctggttgt | 78 |
| *SOX2* | CCACCTACAGCATGTCCTACTCG | GGGAGGAAGAGGTAACCACAGG | 117 |
| *NESTIN* | CTCAGCTTTCAGGACCCCAA | GTCTCAAGGGTAGCAGGCAA | 183 |
| *GLAST* | GGTTGCTGCAAGCACTCATCAC | CACGCCATTGTTCTCTTCCAGG | 95 |
| *BLBP* | GGCTTTGCCACTAGGCAGG | TGACCACTTTGTCTCCTTCTTGA | 76 |
| *GFAP* | GAGGTTGAGAGGGACAATCTGG | GTGGCTTCATCTGCTTCCTGTC | 128 |
| *GLT-1* | TAGCCGCCATCTTTATAGCCC | CGGCTGTCAGAATGAGGAGC | 150 |
| *PLP-1* | GGCCACTGGATTGTGTTTCT | AGGTGGTCCAGGTGTTGAAG | 386-491 |
| *GalC* | GCAACCTCCCGACTTCTAGTA | ACCACTCGTATCCTCGGAAATA | 199 |
| *TBR2 (EOMES)* | CGGCCTCTGTGGCTCAAA | AAGGAAACATGCGCCTGC | 76 |
| *MAPT* | CTCGCATGGTCAGTAAAAGCAA | GGGTTTTTGCTGGAATCCTGGT | 153 |
| *Synaptophysin*  *(SYP)* | CTGCAATGGGTCTTCGCCA | ACTCTCGGTCTTGTTGGCAC | 96 |
| *PILRB* | CCATCAAGGGGACCAAACTCA | TCTGAGTGCCCTTTGCTTTC | 118 |
| *CLU* | CTATCTGCGGGTCACCA C | CTCAGTGACACCGGAAG GAAC | 67 |
| *PLCG2* | CATCCTATATGGCACTC AGTTCG | TCCTGGTGTAAGATTTTC AAGCC | 105 |
| *BAX* | TGGCAGCTGACATGTTTTCTGAC | TCACCCAACCACCCTGGTCTT | 195 |
| *IL-6* | TGCAATAACCACCCCTGACC | TGCGCAGAATGAGATGAGTTG | 104 |
| *IL-8* | AGACAGCAGAGCACACAAGC | ATGGTTCCTTCCGGTGGT | 62 |
| *IL-10* | TGCTGGAGGACTTTAAGGGTTA | GATGCCTTTCTCTTGGAGCTTA | 254 |
| *TNF-α* | CAGCCTCTTCTCCTTCCTGAT | GCCAGAGGGCTGATTAGAGA | 123 |
| *TGF-β* | CGCGTGCTAATGGTGGAAA | CTCGGAGCTCTGATGTGTTGAA | 97 |
| *IL-1β* | AATCTGTACCTGTCCTGCGTGTT | TGGGTAATTTTTGGGATCTACACTCT | 78 |
| *IL-18* | AACAAACTATTTGTCGCAGGAAT | TGCCACAAAGTTGATGCAAT | 72 |
