# Supplementary material for: Advanced patient-specific microglia cell models for pre-clinical studies in Alzheimer’s disease
Source: J Neuroinflammation. 2024 Feb 15;21:50. doi: 10.1186/s12974-024-03037-3 (PMC10870454; doi:10.1186/s12974-024-03037-3)
Supplement: Supplementary file 7 — Additional file 7. Table S2. Correlation analyses between disease-associated phenotypes and APOE genotype identified in MDMi from AD patients. [file 12974_2024_3037_MOESM7_ESM.docx]

**Table S2. Correlation analyses between disease-associated states identified in AD MDMi and *APOE* genotype of AD patients.**

|  | **AD MDMi model** | **Correlation coefficient with**  ***APOE* genotype** | ***P value*** | **Sample size (donors)** |
| --- | --- | --- | --- | --- |
| % contact area between MDMi and ReNcell VM | 3D co-culture | r_s_ = 0.6172 | 0.233 | 6 |
| Number of contacts between MDMi and ReNcell VM | 3D co-culture | r_s_ = 0.1852 | 0.733 | 6 |
| PDGF-AA secretion | 3D co-culture | r_s_ = -0.1826 | 0.778 | 9 |
| EPO secretion | 3D co-culture | r_s_ = 0.2996 | 0.411 | 10 |
| IFN-γ secretion | 3D co-culture | r_s_ = 0.2336 | 0.522 | 10 |
| Angiopoietin 2 secretion | 3D co-culture | r_s_ = 0.4045 | 0.278 | 10 |
| GM-CSF secretion | 3D co-culture | r_s_ = 0.3371 | 0.389 | 10 |
| Total distance surveyed  (around Aβ-FITC) | 3D co-culture | r_s_ = 0.6708 | 0.300 | 5 |
| Speed  (around Aβ-FITC) | 3D co-culture | r_s_ = -0.4472 | 0.600 | 5 |
| %MDMi clustered around Aβ-FITC | 3D co-culture | r_s_ = -0.2236 | >0.999 | 5 |
| IL-6 secretion  (in response to Aβ-FITC) | 3D co-culture | r_s_ = -0.2010 | 0.676 | 7 |
| IL-1β secretion  (in response to Aβ-FITC) | 3D co-culture | r_s_ = 0.2191 | 0.629 | 7 |
| IL-18 secretion  (in response to Aβ-FITC) | 3D co-culture | r_s_ = -0.5578 | 0.210 | 7 |
